# (Patho)Physiology of Glycosylphosphatidylinositol-Anchored Proteins II: Intercellular Transfer of Matter (Inheritance?) That Matters

**DOI:** 10.3390/biom13060994

**Published:** 2023-06-15

**Authors:** Günter A. Müller, Timo D. Müller

**Affiliations:** Institute for Diabetes and Obesity (IDO), Helmholtz Diabetes Center (HDC) and German Center for Diabetes Research (DZD) at the Helmholtz Zentrum München, German Research Center for Environmental Health (GmbH), Ingolstädter Landstraße 1, 85764 Neuherberg, Germany; timo.mueller@helmholtz-munich.de

**Keywords:** adipose and blood cells, diabetes, glimepiride, glycosylphosphatidylinositol (GPI)-anchored proteins (GPI-APs), (G)PI-specific phospholipase C/D (GPI-PLC/D), inheritance of acquired features, protein and information transfer

## Abstract

Glycosylphosphatidylinositol (GPI)-anchored proteins (APs) are anchored at the outer leaflet of the plasma membrane (PM) bilayer by covalent linkage to a typical glycolipid and expressed in all eukaryotic organisms so far studied. Lipolytic release from PMs into extracellular compartments and intercellular transfer are regarded as the main (patho)physiological roles exerted by GPI-APs. The intercellular transfer of GPI-APs relies on the complete GPI anchor and is mediated by extracellular vesicles such as microvesicles and exosomes and lipid-free homo- or heteromeric aggregates, and lipoprotein-like particles such as prostasomes and surfactant-like particles, or lipid-containing micelle-like complexes. In mammalian organisms, non-vesicular transfer is controlled by the distance between donor and acceptor cells/tissues; intrinsic conditions such as age, metabolic state, and stress; extrinsic factors such as GPI-binding proteins; hormones such as insulin; and drugs such as anti-diabetic sulfonylureas. It proceeds either “directly” upon close neighborhood or contact of donor and acceptor cells or “indirectly” as a consequence of the induced lipolytic release of GPI-APs from PMs. Those displace from the serum GPI-binding proteins GPI-APs, which have retained the complete anchor, and become assembled in aggregates or micelle-like complexes. Importantly, intercellular transfer of GPI-APs has been shown to induce specific phenotypes such as stimulation of lipid and glycogen synthesis, in cultured human adipocytes, blood cells, and induced pluripotent stem cells. As a consequence, intercellular transfer of GPI-APs should be regarded as non-genetic inheritance of (acquired) features between somatic cells which is based on the biogenesis and transmission of matter such as GPI-APs and “membrane landscapes”, rather than the replication and transmission of information such as DNA. Its operation in mammalian organisms remains to be clarified.

## 1. Introduction

In eukaryotic cells, a specific class of surface proteins is anchored at the outer leaflet of the phospholipid bilayer of plasma membranes (PMs) via a glycosylphosphatidylinositol (GPI) glycolipid moiety which encompasses about 150, i.e., 0.5–0.8%, of the translated proteins in mammals [1,2] (for a review dealing with the structure and biogenesis of GPI-APs, see [3,4]). One of the major characteristics of GPI-anchored proteins (GPI-APs) is their release from the PMs through a small set of phospholipases of unique substrate but different cleavage specificity (for a review, see [5,6]) rather than a large panel of proteases, each with a different substrate and unique cleavage specificity, as is required for transmembrane proteins (for a review, see [7,8,9,10]). An additional feature is their intercellular transfer, which relies on the complete GPI anchor remaining attached and special carrier mechanisms and structures, among them extracellular vesicles (EVs) and micelle-like complexes. Both attributes alone or in concert may be regarded as the main driving forces for the development and conservation during evolution of GPI-APs vs. transmembrane proteins. The intercellular transfer of GPI-APs with the different structures and molecular mechanisms involved and its putative (patho)physiological implications, which may become cause for the reconsideration of Darwin’s theory of intercellular flow of hereditary information [11], published as Pangenesis theory more than 150 years ago [12,13], are addressed here.

## 2. Transfer via Vesicular Mechanisms

Shortly after the first description of the release of small membrane vesicles (50–100 nm diameter) harboring the transmembrane folate receptor from reticulocytes during their maturation to erythrocytes, subsequently termed exosomes, it has been speculated that their physiological role extends beyond that of mere waste baskets for the removal of inactive or unwanted membrane proteins from the surface of the donor cells [14,15,16,17]. Instead or in addition, exosomes may be engaged in the transfer of membrane and soluble proteins, mRNAs, and miRNAs from donor to acceptor cells as a result of release from, targeting to, and subsequent interaction and fusion with the corresponding PMs. According to this model, the outcome of the intercellular transfer of exosomes is the realization of the information encoded by the transferred materials in the acceptor cells, resulting in the initiation of corresponding (e.g., developmental, growth, metabolic) signaling pathways. One of the first demonstrations that this model for the transfer (of matter and/or information, see Section 7) by exosomes might hold true was the seminal finding that the oncogenic constitutively active version of the receptor tyrosine kinase EGFRvIII becomes transferred from cancerous to normal endothelial cells with resulting downstream angiogenic signaling [18,19,20]. Those findings strongly suggested that oncogene product-harboring tumor-cell-derived exosomes act as information carriers for angiogenesis capable of switching endothelial cells to malignant growth. A large body of additional experimental evidence has accumulated so far, dealing mainly with the transfer of the cancer phenotype [21,22], and is compatible with the meanwhile broadly accepted role of exosomes in intercellular information transfer and communication, relevant for tumor development and metastasis (for a review, see [19,23,24,25,26]). Subsequently, exosomes have been attributed a variety of additional functions, among them secretion and immunomodulation [27], inflammation and coagulation [27,28], glucose and lipid metabolism [27,29,30], developmental and reproductive biology [31], drug resistance [23], and other (patho)physiological processes (for a review, see [32,33,34,35,36]).

In the following period it was demonstrated that the intercellular transfer of matter through exosomes not only encompasses their transmembrane proteins and soluble contents, but also holds true for GPI-APs embedded in the outer leaflet of their phospholipid bilayer. Among the first GPI-APs shown to be transferred by exosomes were CD62, CD55, CD59, CDw52, and CD73. They are all synthesized and released into exosomes by cells of the male genital tract rather than by the spermatozoa themselves, then transported via fluid secretions and finally inserted into the PMs of the spermatozoa [31,37]. Exosomes containing those GPI-APs account for a considerable portion of the structural alterations and acquisition of novel functional properties at the surface of spermatozoa during their maturation and may support their protection from immune attack in the male as well as female reproductive tracts (for a review, see [38]). Furthermore, prostasomes isolated from human seminal plasma, which represent specialized exosomes assembled, stored, and released by the glandular epithelial cells of the prostate [39], have been demonstrated to transfer CD59 to the spermatozoa of human patients suffering from paroxysmal nocturnal hemoglobinuria (PNH) and rabbit erythrocytes [40]. In agreement, prostasomal CD59, which is released by prostate cancer cells to a considerably higher degree than by normal cells, was shown to be transferred to rabbit erythrocytes (lacking CD59) in vitro and to foster inhibition of complement (C5b-9)-mediated lysis more efficiently in comparison to that released from non-malignant cells [41]. Consequently, it has been proposed that the intercellular prostasomal transfer of CD59 can protect autologous and allogenic cells from complement attack in the genital tracts. In addition to prostasomes, the transfer of GPI-APs to spermatozoa via luminal fluids has been reported to happen in male and oestrous female reproductive tracts and attributed to exosomes released into the epididymis, so-called epididymosomes. Moreover, exosomes, coined as uterosomes, have been detected in murine oestrous female reproductive fluid and found to harbor the GPI-AP sperm adhesion molecule 1 (SPAM1/PH-20) at their surface, and consequently, to exert hyaluronidase activity in male and female fluids [42]. Upon incubation, SPAM1 with the full-length GPI anchor was transferred from both epididymosomes and uterosomes to the PMs of caudal spermatozoa with accompanying transient interaction of the exosomes and PMs, and was finally placed at the acrosome and midpiece of the flagella [43]. This implies that those interactions represent a necessary mechanistic step of the exosome-mediated transfer of GPI-APs from murine reproductive fluids to sperm. Hypothetical models for the molecular mechanisms engaged in the transfer of GPI-APs from seminal exosomes to the surface of spermatozoa during their maturation as well as in course of maintenance of their function have been presented [44] (for a review, see [45]).

PNH is a hemolytic disease caused by a somatic mutation in the PIG-A gene. The resulting defect in the synthesis and cell surface expression of GPI-APs in the affected hematopoietic cells leads to elevated sensitivity of the erythrocytes to complement-mediated lysis. Importantly, the release of CD55 and CD59 into exosomes during maturation of reticulocytes and their transfer to GPI-deficient erythrocytes of PNH patients has been demonstrated both in vitro [46] and in vivo [47]. One of the first studies using a recombinant fusion protein-GPI for studying the intercellular transfer of GPI-APs was performed with CD4-GPI. Upon adenoviral overexpression in HeLa cells, it was transferred to the non-infected parental cells during co-culture [48]. Blockade of CD4-GPI transfer by separation of the co-cultivated cells using a membrane impermeable for vesicular structures led to the conclusion that transfer of GPI-APs in general is predominantly mediated by exosomes rather than aggregates [49] and other non-vesicular structures such as micelle-like complexes. Only later this argumentation turned out to be incorrect (see Section 3).

The hypothesis that GPI-APs transferred via exosomes manage to exert clear-cut changes in the (patho)physiological state of the acceptor cells in general, was subsequently extended to metabolic and signaling pathways in particular. In greater detail, a novel regulatory mechanism of lipid metabolism in rat adipocytes was elucidated, which was induced by a variety of lipogenic stimuli such as palmitate, H_2_O_2_, the antidiabetic sulfonylurea (SU) glimepiride, and insulin-mimetic phosphoinositolglycans (PIGs), and depends on the exosomal transfer of GPI-APs. The underlying molecular processes can be summarized as follows: (i) release of exosomes harboring GPI-APs, among them the (c)AMP-binding protein as well as cAMP-degrading phosphodiesterase Gce1, and the AMP-degrading 5′-nuceotidase CD73 from donor adipocytes [50,51], (ii) targeting of and interaction with acceptor adipocytes of the released exosomes [52], (iii) fusion of the exosomal membranes and acceptor adipocyte PMs [53], (iv) translocation of Gce1 and CD73 from the acceptor adipocyte PMs to intracellular lipid droplets (LDs) [54,55], and (v) hydrolysis of (c)AMP at the LD surface zone by the concerted actions of Gce1 and CD73 [56]. This series of events ensures the coordinated stimulation of the esterification of fatty acids into, and concomitant inhibition of, their liberation (by hormone-sensitive lipase and adipocyte triglyceride lipase) from neutral lipids in the acceptor adipocytes. This ultimately leads to lipid storage and size gain of adipocytes. Both Gce1 and CD73 apparently operate as the critical exosomal components which are transferred from donor to acceptor adipocytes. Remarkably, Gce1 and CD73, which typically reside at lipid rafts of PMs, i.e., liquid-ordered domains of high concentrations of cholesterol and (glyco)phospholipids with long-chain saturated fatty acids, and in exosomes, have been detected to be transiently expressed at the surface of cytoplasmic LDs, in addition [57]. This atypical location of a GPI-AP results from their translocation from PM lipid rafts, which apparently involves exosomes as well [58]. The residence at LDs of Gce1 and CD73 is presumably guided by the insertion of their GPI anchor fatty acyl chains into the phospholipid monolayer of LDs. This monolayer typically covers the neutral lipid core and contains specific LD-associated structural proteins (e.g., perilipin-A) and enzymes (e.g., hormone-sensitive lipase) (for a review, see [59,60,61,62,63]). Thus, LDs represent a perfect environment for displaying full-length GPI-APs at their surface through insertion of their anchor into the phospholipid monolayer. The apparent translocation of GPI-APs from PMs to cytoplasmic LDs in adipocytes may represent a mechanism leading to the incorporation of GPI-APs onto the surface of other lipophilic particles such as surfactant-like particles (SLPs), milk fat globules (MFGs), and nodal vesicular particles (NVPs) (for a review, see [6,27]). It remains to be clarified whether the localization GPI-APs at SLPs and MFGs is restricted to their surface, i.e., the outer leaflet of the phospholipid bilayer, or also holds true for the phospholipid monolayer of their lipophilic core, in addition.

**Figure 1:**
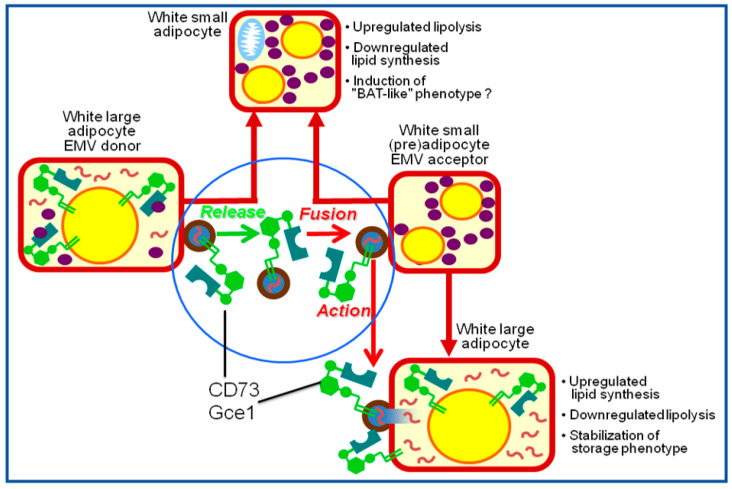
Model for the involvement of interadipocyte material transfer with regard to the GPI-APs Gce1 and CD73 for the regulation of lipid metabolism, storage, and cell size within adipose tissue depots. The model is based on a polarized structure of adipose tissue depots with large mature adipocytes harboring a single or a few LD(s) only. These are capable of very pronounced esterification in parallel with high lipolysis and located in the immediate vicinity of blood vessels, where they form a gradient together with nascent adipocytes of small and very small size. Prolonged delivery of free fatty acids by the blood vessels and their resulting efficient esterification into LDs will cause the size increase of the adipocytes and exhaustion of their lipid storage capability. This will lead to the upregulation of the lipolytic release of fatty acids from their LDs into the interstitial space. This will only trigger the maturation of (very) small precursor adipocytes lacking visible LDs in the deeper adipose tissue layers that are capable of esterification and lipolysis to a very moderate degree by one or several types of intercellular signaling. Upon induction of novel and specific cell-to-cell contacts, as are already formed between the large adipocytes, or the (vesicular) secretion of adipokines [67] such as acylation stimulation protein ASP [68], or soluble factors such as 15-keto-PGE2 [69,70], the targeted cell adhesion molecules or adipokine receptors at the cell surface or nuclear hormone receptors, respectively, induce the signaling for stimulation of the esterification and/or inhibition of lipolysis. This in concert finally leads to upregulated LD biogenesis in the growing small adipocytes. Alternatively, or in addition, upon being challenged with high concentrations of fatty acids originating directly from the blood vessels or from lipolytic release in response to physiological or pharmacological stimuli such as hydrogen peroxide or the anti-diabetic sulfonylurea drug (SU) glimepiride [71], the large adipocytes release Gce1 and CD73 into EVs. Following passage through interstitial tissue spaces and eventually thereafter through the circulation, the EVs interact with the PMs of (very) small adipocytes for subsequent transfer of Gce1 and CD73 to the surface of LDs. Upon arrival, these enzymes degrade (c)AMP. Again, the resulting parallel upregulation of esterification and downregulation of lipolysis lead to the enlargement of the LDs and size of the adipocytes.

The size of mammalian adipocytes is critically determined by the filling state of their LDs, which considerably differs between large and small cells within the same adipose tissue depot. The role of GPI-AP-harboring exosomes in the coordination of LD biogenesis between differently sized adipocytes was investigated with mixed populations of small and large isolated rat adipocytes as well as native adipose tissue pieces from young and old rats [64]. The analysis revealed that large adipocytes were more potent in releasing CD73 into exosomes and less potent in translocating CD73 to LDs in comparison to small adipocytes. Furthermore, mixed populations of small and large adipocytes were found to be more active in LD biogenesis upon lipogenic stimulation than either small or large adipocytes. Most importantly, it was demonstrated that upregulation of LD biogenesis in both adipocytes and adipose tissue pieces from young rats as well as esterification stimulation were inhibited by the depletion of CD73-harboring exosomes from the incubation medium and extracellular spaces, respectively [65]. It was concluded that adipocytes of different sizes within the same adipose tissue depot coordinate the stimulation of their lipid synthesis and LD biogenesis via the release of GPI-APs from large cells, leading to subsequent transfer via exosomes to small cells and final translocation to the LDs of small cells. This transfer of matter via exosomes harboring GPI-APs may shift the burden of triacylglycerol storage from large to small adipocytes [66]. Importantly, in contrast to the pathophysiological role of the intercellular transfer of transmembrane proteins via exosomes, as described above with EGFRvIII, the transfer of LD biogenetic and antilipolytic materials via exosomes between large and small adipocytes within the same adipose tissue depots represents an example of a physiological and normal regulatory function of exosomes. A hypothetical model for the (patho)physiological role of the exosome-mediated transfer of GPI-APs between adipocytes is presented (Figure 1).

However, it remains to be clarified whether exosomes released from large adipocytes remain captured within the corresponding adipose tissue depot or are able to leave it and enter the circulation via passage across adipose tissue vascular endothelial cells. If exosomes were entrapped in the releasing adipose tissue depot, the intercellular transfer of GPI-APs would be restricted to cells (adipocytes, vascular stromal and endothelial cells, macrophages) of this depot. In this regard, the physiological insults of transferred Gce1 and CD73 may differ between the various adipose tissue cell types with the coordinated stimulation of LD biogenesis and inhibition of lipolysis occurring only in adipocytes. In case of the spreading of exosomes harboring GPI-APs, among them Gce1 and CD73, via the circulation, “non-physiological” acceptor cells such as myocytes, hepatocytes, and pancreatic β-cells may be forced to synthesize and store neutral lipids in LDs. This phenomenon is known to lead to deleterious “lipotoxic” consequences such as impaired insulin stimulation of glucose and lipid metabolism (insulin resistance) and defective insulin secretion (ß-cell failure). The reason for this is that the corresponding insulin target and releasing cells, at variance with adipocytes, are not equipped with the molecular machineries to efficiently store and mobilize fatty acids. There is convincing experimental evidence that lipid storage at excessive amount and at inappropriate location ultimately interferes with the physiological functions of tissue cells other than adipocytes. Consequently, “lipotoxic” mechanisms have meanwhile been accepted to be causally involved in the development of type 2 diabetes and the metabolic syndrome (for a review, see [72,73,74,75,76,77]).

It is conceivable that the cell surface expression of GPI-APs leads to changes in the physicochemical characteristics of PMs which drive the release and/or transfer of GPI-APs, among them GPI-anchored (co-)receptors and other relevant signaling components, capable of triggering specific signal transduction processes. In fact, some data argue for this possibility: (i) The GPI-AP Cripto-1 (CR-1), known to operate as an obligate co-receptor of the TGFß family ligand Nodal [78], has been demonstrated to exert its paracrine signaling function only if associated with vesicles but not in soluble form [79]. Intercellular transfer was strictly dependent on the formation of unique extensions of the PMs by CR-1-expressing cells, which was more pronounced than that by control cells [80]. (ii) The GPI-AP human NKG2D ligands UL16 binding proteins (ULBP) 1-3 have been shown to be transferred between natural killer cells with rapid kinetics and at different efficacies [81]. Transfer efficacy was dependent on the nature of the NKG2D ligands, the receptor-ligand interaction and the state of immune activation of the natural killer target cells, and was correlated to their degranulation [81]. (iii) The transferred NKG2D ligands bound to ULBP1-3 were found to upregulate the activation of autologous natural killer cells. Unexpectedly, re-transfer of the transferred ligand-receptor complexes to autologous effector cells caused their death [81]. It was concluded that the exosomal intercellular transfer of GPI-APs can proceed in both directions in general, and that it exerts a prominent role in the homeostatic downregulation of the immune response in natural killer cells in particular.

## 3. Transfer via Non-Vesicular Mechanisms

The first report about the transfer of GPI-APs in vitro was published before the elucidation of the chemical composition and structure of GPI anchors in 1985 [82,83]. In 1977, Huestis and coworkers found that a subset of membrane proteins, localized at the extracellular leaflet of the erythrocyte PMs, such as the GPI-AP acetylcholinesterase (AChE), is selectively and reversibly transferred from erythrocytes to liposomes [84,85,86]. Subsequently, the purified human erythrocyte GPI-AP decay accelerating factor (DAF or CD55) has been demonstrated to associate with sheep erythrocytes upon incubation, and concomitantly to maintain its biological activity, i.e., to block human complement attack and the resulting erythrocyte lysis [87,88]. Following the identification and structural characterization of GPI anchors in 1985, additional experimentation revealed the transfer of GPI-APs from donor to acceptor PMs or liposomes as well as the insertion of enriched or purified detergent-solubilized GPI-APs into acceptor PMs. Each of these processes is presumably accompanied by the direct intercalation of the GPI anchors into the outer leaflet of the PM or liposomal phospholipid bilayer [85,86].

One of the first demonstrations of a recombinant fusion protein-GPI to be transferred between cultured cells by non-vesicular mechanisms was performed with CD4-GPI. This fusion protein became transferred in a biologically active state between adeno-associated virus-transduced HeLa cells or from those to GPI-deficient human erythrocytes upon direct cell contact or exposure to HeLa cell supernatants which lacked any vesicular structures [48,49] (for a review, see [89]). In parallel, GPI-linked complement restriction factors were found to be transferred in vivo from the erythrocyte PMs derived from transgenic mice to endothelial cells in functional form [90] (for a review, see [91,92]).

The validity of this approach was reinforced with biotinylated human DAF55 and AChE as well as murine GPI-anchored B7-1 and B7-2 for determination of their distribution and signaling function upon insertion into the PMs of HeLa or Chinese hamster ovary cells [93]. Three modes of association with the cell surface were observed for each reporter GPI-AP, (i) unspecific adsorption and/or peripheral embedding, (ii) insertion into non-lipid raft membrane areas, and (iii) insertion into lipid rafts. Movement from the former two localizations to the latter was found to occur, however, only at slow rate. It led to the acquisition of the native function, i.e., inhibition of the complement-induced formation of the membrane attack complex (MAC) and corresponding enzymic activity, respectively. These data argued for conservation of the native function of exogenously inserted GPI-APs and thus for the usefulness of cell surface engineering/painting with GPI-APs, for instance, with modified cancer cell antigens of tumor cells for the treatment of cancer (see Section 4).

Those cellular studies on GPI-AP transfer were supplemented by cell-free and liposomal test systems. An important example is the use of monolayers of phosphatidylcholine at the air–water interface as model membranes which were incubated with the GPI-AP bovine intestinal mucosa alkaline phosphatase (AP). It was found that phospholipids with unsaturated fatty acids at the acceptor cell PMs considerably reduced the interface interaction forces elicited by the insertion of the GPI-APs [94]. This feature of the interaction between GPI-APs and phospholipids may explain the differential targeting of GPI-APs to lipid raft vs. non-raft domains of the acceptor PMs. Erythrocyte-liposome systems have also been used to address the question as to whether the transfer of GPI-APs between membranes operates spontaneously or is supported by some catalyst(s). Surprisingly, detailed analysis revealed that the GPI-AP AChE is transferred from erythrocytes to both small light and large heavy vesicles, which are both shed from the erythrocytes upon exposure to liposomes, rather than to the liposomes themselves [95,96]. This finding demonstrated that liposomes cause the release of AChE from erythrocytes into large heavy vesicles, which could be identical with microvesicles, with subsequent transfer to and accumulation in the small light vesicles. The results strongly suggested that GPI-APs do not spontaneously transfer between the PMs of mammalian cells [97,98]. Consequently, it has been concluded that the transfer of exogenously added (native) GPI-APs or recombinant fusion proteins-GPI critically depends on the activity of a still unknown catalyst. If valid, this entity may be functional at the acceptor PMs, and is apparently expressed in those acceptor cells, which have been used for the so-called “cell surface painting or engineering” so far (for a review, see [99,100]).

A number of findings on GPI-AP transfer took advantage of studies on PNH. This acquired disease is caused by defective synthesis of precursors for GPI anchors in the stem cells of the blood cell lineage. This leads to erythrocytes devoid of GPI-APs, among them the complement regulatory/inhibitory components CD55, CD59, and the homologous restriction factor (HRF). GPI-deficient blood and immune cells are therefore extremely sensitive towards attack and lysis provoked by the autologous complement system (for a review, see [101,102]). Importantly, it has been demonstrated that GPI-deficient erythrocytes from PNH patients (re-)gained considerable resistance towards complement-mediated destruction by incubation with purified CD55 [103], CD59 [104], or HRF [105]. Apparently, these GPI-APs are transferred to and exert their biological activity at the PNH erythrocytes upon their insertion into the outer PM leaflet in correct orientation. On the basis of the demonstrated transfer of full-length and functional CD55 and CD59 from erythrocytes to endothelial cells in transgenic mice as well as under physiological conditions such as organ and bone marrow transplantation, it has been proposed that, in general, cells with limited or missing capacity for the biogenesis of GPI-APs such as erythrocytes, endothelial cells and spermatozoa may receive them under (patho)physiological conditions or be “painted” by them under artificial conditions upon transfer from GPI-AP-expressing donor cells [89,90].

Mammalian GPI-APs are known to transmit signals across PMs upon their crosslinking by antibodies (for a review, see [106,107]). Immediately after the transfer of rat brain Thy-1 to mouse lymphocytes [108] or sheep CD59 to human neutrophils [109], both GPI-APs failed to efficiently signal across PMs. However, human CD59 inserted into U937 monocytes triggered downstream signaling, as reflected in the stimulation of intracellular Ca^2+^-fluxes, upon prolonged incubation which resulted in its redistribution into PM lipid rafts [110]. This led to the conclusion that GPI-APs transferred to the PMs of acceptor, here immune, cells and following their redistribution to their preferred residence, the lipid rafts, obtain their, here Ca^2+^-triggered, signaling competence. Additional findings strongly indicate that GPI-APs are required for normal immune cell, in particular, lymphocyte, function and maintenance of normal peripheral immunological, in particular, lymphoid, homeostasis rather than for immune cell, in particular, lymphocyte, development (for a review, see [106]).

Transfer of GPI-APs into the outer leaflet of the PM bilayer seems to be mediated by their long-chain fatty acyl chains on the basis of the following findings: (i) It is strictly dependent on the diacylglycerol or phosphatidate moiety of the GPI anchor [111]. (ii) It is more efficient with GPI-APs equipped with GPI anchors harboring two fatty acyl chains compared to a single acyl moiety [111]. (iii) It is hardly affected by treatment of the acceptor PMs with proteinase K or sialidase before [112] as well as high salt treatment following incubation with the GPI-APs [87]. This argues against their peripheral association with proteinaceous or lipidic cell surface components. (iv) It is more efficient at higher temperatures [87,113,114,115], which may be due to increased fluidity of the PMs as well as lateral mobility of their components with the resulting inclusion of GPI-APs into specific PM domains, such as the lipid rafts. (v) It is inhibited by lipid- and fatty-acid-binding proteins such as albumin, or lipoproteins such as human high and low density lipoproteins (HDL, LDL), which, however, failed to extract GPI-APs following their transfer [87,115]. Importantly, in the above studies, the purified GPI-APs were transferred into the PMs of intact acceptor cells in the absence of detergent [107,115] or in the presence of very low concentrations of detergent under preservation of the structural intactness of the PMs [87,111].

The following different molecular mechanisms have been proposed for the insertion of GPI-APs into acceptor membranes or cells: (i) Insertion as monomers from stabilizing detergent micelles or from lipoproteins or from lipid- or fatty-acid-binding proteins upon their dissociation. (ii) Insertion as homo-oligo/multimers, which are generated upon removal of detergent in the course of the purification of GPI-APs, following their contact with hydrophobic membrane domains such as lipid rafts. (iii) Insertion following constitutive endocytosis of the homo-oligo/multimers of GPI-APs, and their subsequent dissociation into monomers in the endosomal compartment. Upon their transfer to the luminal leaflet of the endosomal membranes and final vectorial vesicular transport to the cell surface, the GPI-APs become expressed at the extracellular leaflet of the PMs. All these hypothetical mechanisms are compatible with the insertion of the GPI anchor fatty acyl chains either directly into lipid rafts containing high concentrations of saturated long-chain glyco(sphingo)lipids and cholesterol or into non-raft domains with distribution all over the cell surface in random fashion and subsequent lateral diffusion into lipid rafts. Both pathways would guarantee the acquisition of the typical topological and functional (e.g., signaling) characteristics of the transferred GPI-APs.

Native GPI-APs are targeted to their specific (sub)cellular localizations in course of their biosynthesis, leading to their characteristic distribution within PMs (e.g., apical vs. basolateral) and between PMs and intracellular membranes (e.g., Golgi apparatus, endoplasmic reticulum) as well as between distinct cells and tissues (for a review, see [6]). This raised important questions as to whether the transfer of GPI-APs occurs in vivo and, if so, whether it could lead to their aberrant (inter)cellular distribution, possibly under certain (patho)physiological conditions. About three decades ago, the transfer of GPI-APs in vivo was reported for the first time [90,116,117]. However, since those studies dealt with GPI-anchored serum complement regulatory proteins exclusively, the second question has remained unanswered. In fact, a concentration gradient of GPI-APs between donor and acceptor cell PMs could facilitate their transfer between neighboring cells of the same tissue depot. Alternatively, it is conceivable that fatty-acid- or lipid-binding serum proteins mediate transfer through shielding of the GPI anchor from access of the aqueous milieu of blood and other body fluids. Those proteins could simultaneously catalyze the insertion step into the acceptor PM outer leaflet [97]. HDL has been suggested as candidate for a lipid-binding serum protein. It may exert this role on the basis of its reported interaction with CD59 in human serum [118] and its ability to stimulate the transfer of GPI-APs between PMs under serum-free conditions [119]. However, at present it cannot be excluded that serum lipoproteins with loaded GPI-APs are endocytosed upon binding to their cognate receptors according to the above mechanism (iii) for their trafficking to the cell surface. Moreover, the demonstrated in vivo transfer of GPI-APs from erythrocytes could have involved EVs since those are known to be released from erythrocytes in response to mechanical stress, age, prolonged storage, ATP depletion [120], or contact with other membranes [97,98]. After targeting and interacting with the acceptor PMs, EVs could mediate the direct insertion of the loaded GPI-APs, directly fuse with the acceptor PMs, or be endocytosed and recycled back to the cell surface. The operation of one or the other of these putative mechanisms of non-vesicular transfer of GPI-APs in vivo under (patho)physiological conditions remains to be demonstrated.

In one of the first detailed biochemical and microscopic characterizations of non-vesicular structures mediating intercellular transfer, the GPI-APs CD59, CD55, and CDw52, but not the transmembrane protein CD46, were identified in high-speed supernatants of seminal plasma as stable high-molecular aggregates of high buoyant density. Those GPI-APs were found to be transferred in vitro from this prostasome- and vesicle-free fraction to spermatozoa, where they may contribute to the creation of new surface properties, among them resistance towards complement attack in the seminal plasma [121,122,123].

Convincing experimental data have revealed lipoproteins as candidate proteins facilitating the intercellular non-vesicular transfer of GPI-APs. They may interact with (presumably fatty acyl chains of their) GPI anchors in general [124,125,126,127,128], and with that of clusterin and apolipoprotein J in particular [129,130]. These fatty acyl or phospholipid carriers, which are abundantly expressed in epididymal and uterine luminal fluids, were found to produce monomers of the GPI-AP sperm adhesion molecule 1 (SPAM1) from its transfer-incompetent homo-oligomeric aggregates. Those monomers, stabilized by hydrophobic interactions, are then destined for transfer by a non-vesicular mechanism from the soluble fraction of the luminal fluids to human and mouse sperm membranes [129,130]. Consequently, a lipid-exchange model relying on phospholipid carriers such as clusterin was presented to explain the transfer of GPI-APs to the sperm surface [129,131]. Prior to these findings, the transfer of *Schistosoma mansoni* and *Trypanosoma brucei* GPI-anchored glycoproteins such as VSG221 to serum lipoproteins of infected patients suffering from chronic schistosomiasis and sleeping sickness, respectively, has been reported [124]. Endocytosis of the lipoprotein-GPI-AP complexes by host cells carrying the immunoglobulin-F_c_ receptor were thought to provoke their clearance by immune cells, disruption of lipid homeostasis, and lipid accumulation in neutrophils with accompanying apoptosis. This sequence of events may ultimately end in the recycling of the GPI-APs to the host cell surface with aberrant antigen presentation and, consequently, insufficient immune response towards the pathogen [124].

One of the first reports that non-vesicular transfer of GPI-APs is a regulated process dealt with the GPI-APs cellular prion protein (PrP^C^) and CD90 which were both transferred from a PrP^C^ expressing neuroblastoma cell line to human erythroleukemia cells (ELCs) at low efficacy [132]. Importantly, transfer, which required the complete GPI anchor and direct cell-to-cell contact (see Section 5), was upregulated by incubation of both donor and acceptor cells with the protein kinase C activator phorbol 12-myristate 13-acetate. Apparently, intercellular transfer of a subset of GPI-APs is under control of signaling by protein kinase C.

## 4. Transfer via Micelle-like Complexes

In mammalian organisms, full-length GPI-APs can be released from donor cells and tissues into extracellular compartments such as interstitial fluids and the blood stream in response to endogenous or exogenous cues and incorporated together with (lyso)phospholipids and cholesterol into micelle-like complexes (for a review, see [6]). Those GPI-APs may escape degradation by serum GPI-specific phospholipase D (GPLD1) and then be transferred to acceptor blood and tissue cells. To study this possibility in vitro, the interaction with GPI-APs and activity of GPLD1 and the transfer of the GPI-APs were analyzed using chip-based biosensing with horizontal surface acoustic waves (SAWs) of reconstituted micelle-like GPI-AP complexes [133]. For this, a chip- and microfluidic channel-based biosensor was developed, which monitors any interaction at the chip surface by changes in the phase and amplitude of the horizontal SAWs propagating over the chip surface. Upon measurement, the complexes were identified in rat as well as human serum as changes in SAW phase shift, which reflected the amount of full-length GPI-APs in complex with lipids, as well as in SAW amplitude, which reflected the viscoelasticity of the complexes (for a review, see [6]).

Both rat and human serum GPLD1 activity was found to be positively correlated to the hyperglycemic/hyperinsulinemic state of the donor organisms which was primarily caused by the upregulated interaction of the GPLD1 with micelle-like GPI-AP complexes [133]. Other serum proteins also managed to interact with the phosphoinositolglycan (PIG) portion of the GPI anchors. Importantly, upon incubation, full-length GPI-APs were transferred from micelle-like complexes and, with lower efficacy, from reconstituted HDL and liposomes to acceptor adipocytes. The accompanying increases of lysis of the adipocytes, as monitored by lactate dehydrogenase release, as well as of GPI-AP transfer, were diminished by catalytically inactive but binding-competent GPLD1 and other serum proteins, in positive correlation to the hyperglycemic/hyperinsulinemic state of the donor rats and diabetic state of the probands, respectively [133]. On the basis of these results, it was concluded that full-length GPI-APs, released into the circulation from metabolically dysregulated donor cells, exert deleterious effects upon transfer to acceptor cells unless their GPI anchor becomes bound to the GPLD1 or other serum proteins. Those become upregulated in diabetic rats and humans, and thereby prevented GPI-APs from being transferred to blood and tissue cells. The observed correlations of the transfer of GPI-APs through non-vesicular mechanisms with the metabolic state [133] and age [134] justified efforts for the identification of the complexes of GPI-APs and serum proteins for the prediction and stratification of metabolic diseases such as obesity and diabetes.

For this, the involvement of micelle-like GPI-AP complexes in the transfer of GPI-APs between donor and acceptor PMs was tested using the chip-based SAW biosensor in a cell-free configuration with covalently immobilized acceptor PMs (from rat and human adipocytes and erythrocytes) [135]. The transfer of GPI-APs to the acceptor PMs was monitored upon injection into the chips of donor erythrocyte or adipocyte PMs and then of antibodies against GPI-APs as a phase shift of the horizontal SAWs induced by antibody binding to the transferred GPI-APs. Transfer was found to depend on time and temperature, and to be restricted to GPI-APs (i.e., no transfer of transmembrane proteins was detected with the SAW biosensor). It was inhibited by adsorption of GPI-APs to a GPI-binding toxin or injection of serum proteins into the chip channels with accompanying accumulation in the channels of non-vesicular structures consisting of full-length GPI-APs in association with (lyso)phospholipids and cholesterol. Transfer was restored in concentration-dependent fashion by synthetic PIGs mimicking the glycan core of GPI anchors. Interestingly, the metabolic state (genotype and feeding state) of the rats which served as sources for the PMs and sera was correlated to the efficacy of transfer between the adipocyte and erythrocyte PMs and its blockade, respectively, being highest for obese diabetic rats [135]. The data obtained with the cell-free chip-based biosensor suggested that transfer of full-length GPI-APs operates between isolated PMs, involves micelle-like complexes, and depends on some biophysical characteristics of the donor and/or acceptor PMs, which are determined by the metabolic state of the corresponding cells.

After having elucidated the transfer of GPI-APs from isolated donor PMs via micelle-like complexes to isolated acceptor PMs and its correlation to the metabolic state of the corresponding donor cells, the possibility of transfer between intact donor and acceptor cells with accompanying impact on the metabolism of the latter was investigated [136]. Using transwell co-cultures and chip-based biosensing of PMs prepared from the acceptor cells, for the amount of GPI-APs, full-length GPI-APs were found to be transferred from micelle-like complexes or human ELCs or from donor adipocytes to GPI-deficient acceptor adipocytes or ELCs. Transfer was accompanied by stimulation of glycogen (ELCs) and lipid (adipocytes) synthesis. An increase in transfer as well as metabolism was counteracted by addition of serum proteins, GPLD1, albumin, or bacterial phosphatidylinositol-specific phospholipase C (PI-PLC) for the removal of total GPI-APs from the culture medium. Serum blockade of transfer in parallel of glycogen/lipid synthesis, which was most pronounced for rats of dysregulated metabolic states or hyperinsulinemic/hyperglycemic human probands, was abrogated by synthetic PIGs which cause the displacement of GPI-APs from the serum proteins. Moreover, the combination of large donor and small acceptor rat adipocytes led to maximal upregulation of transfer and lipid synthesis [136]. These results argue for the transfer of full-length GPI-APs between adipocytes and blood cells in both homologous and heterologous configuration. As has been previously demonstrated for isolated PMs [135], serum proteins, in correlation to the metabolic state of the donor organisms, and PIGs, in correlation to their structural similarity to the glycan core, caused the inhibition and restoration, respectively, of transfer as well as lipid and glycogen synthesis. Thus, GPI-APs are transferred between intact cells under involvement of micelle-like complexes. The resulting upregulation of lipid and glycogen synthesis in the acceptor cells and its dependence on the size and metabolic state of the cells such as lipid loading of the adipocytes, argue for the (patho)physiological relevance of the intercellular GPI-AP transfer in general, and its role in the “direct” short-distance control of metabolism in particular.

A working model for the “direct” physiological mode of (“vertical” and “horizontal”) transfer of GPI-APs over short distance between human adipocytes and from human adipocytes to blood cells and stimulation of lipid droplet and glycogen granule biogenesis, respectively, with the aid of transwell co-culture, in particular, has been presented (see reproduction from [136] in Supplementary Materials, Figure S1). It depicts the transfer of full-length GPI-APs from donor to acceptor cells within the same tissue depot and from donor tissue cells to acceptor blood cells in the immediate vicinity and its regulation by serum proteins and the metabolic state of the mammalian organism. The main conclusions are as follows (numbering of the distinct steps as shown in Supplementary Materials, Figure S1): Full-length GPI-APs are released from the outer leaflet of the PM bilayer of large lipid-loaded human adipocytes (panel b, upper compartment, “direct” transfer) into the interstitial spaces of adipose tissue depots (1). Specific characteristics of the adipocyte PMs (e.g., viscoelasticity, stiffness), which have been found to be correlated to the age and metabolic state of both rat and human adipocytes [137,138,139,140], may support release. Released GPI-APs are inserted into the outer leaflet of the PM bilayer of small (pre)adipocytes, which contain only small lipid droplets and few GPI-APs and reside near to the large adipocytes within the same tissue depot (2). The resulting increase in the number of GPI-APs at PMs (3) causes the upregulation of lipid synthesis (4) as well as lipid droplet biogenesis with accompanying conversion of small into large adipocytes (5). Depletion of serum GPI-binding proteins from the adipose tissue depots, putatively as a consequence of the continuous blood stream and/or elimination by the liver, drives physiological, i.e., “direct” (transfer without involvement of GPI-binding protein), “vertical” (transfer between the same cell type), and “downward” (transfer from old to young cells) transfer of GPI-APs. As the final physiological outcome of this type of GPI-AP transfer, the shifting of the burden of lipid droplet biogenesis and lipid storage from large to small adipocytes within the same tissue depot seems to represent a sound candidate.

At variance, for the pathophysiological, i.e., “indirect” (under involvement of GPI-binding proteins) and “horizontal” (between different cell types) transfer of GPI-APs from large adipocytes, which contain a single large LD and many GPI-APs, to distant blood (or non-adipose tissue) cells to happen, the released full-length GPI-APs have to be transported across endothelial cells into the blood (6). However, serum GPI-binding proteins (7), among them albumin (8) and catalytically inactive GPLD1 (absence of divalent cations) (9), recognize the glycan core of the GPI anchor. At contrast, active GPLD1 (presence of divalent cations) degrades the GPI anchor (10) with concomitant generation of inositolglycan-(IG-)proteins from the full-length GPI-APs (11). In consequence, pathophysiological transfer is prevented by both types of interaction of serum proteins and GPI-APs (8–10), each blocking their insertion into PMs (12) and the accompanying biogenesis of glycogen granules in blood (and non-adipose tissue) cells (13). At variance, pathophysiological “indirect” transfer becomes enabled by the presence of PIG compounds in the blood (14). This leads to displacement of full-length GPI-APs from the serum GPI-binding proteins (15). The displaced GPI-APs may be arranged in (lyso)phospholipid-harboring micelle-like complexes or in (homo- or heteromeric) aggregates during passage across the interstitial spaces of adipose tissue depots and the bloodstream (not depicted in this model), as have been characterized previously [141,142]. Thereafter, those GPI-APs insert into the outer leaflet of blood (and non-adipose tissue) cell PMs (16) under parallel upregulation of glycogen granule biogenesis (17). In conclusion, the concerted and coordinated action of serum GPI-binding proteins and PIGs seems to play a critical role for the differential routing of the released full-length GPI-APs into the physiological “direct” vs. the pathophysiological “indirect” mode of their transfer.

Next, it has been demonstrated that the “indirect” transfer of GPI-APs to as well as the induction of the anabolic phenotype in the acceptor cells, as reflected in the stimulation of glycogen synthesis and granule biogenesis in the GPI-deficient acceptor ELCs in the transwell co-culture, is under the control of certain hormones and drugs [143]. Insulin and the anti-diabetic SU glimepiride, which has been shown to stimulate glucose transport and metabolism in insulin target cells and tissues independent of insulin both in vitro [144] and in vivo [145], induced the concentration-dependent reduction of both transfer and anabolic effect [143]. Unexpectedly, it became evident that the insulin- and glimepiride-induced blockade of GPI-AP transfer as well as glycogen synthesis is abrogated by rat serum in a volume-dependent manner. The restoration of both transfer and anabolic effect was found to depend on the metabolic state of the rats as serum donors, with serum from hyperinsulinemic hyperglycemic rats being most potent [143].

**Figure 2:**
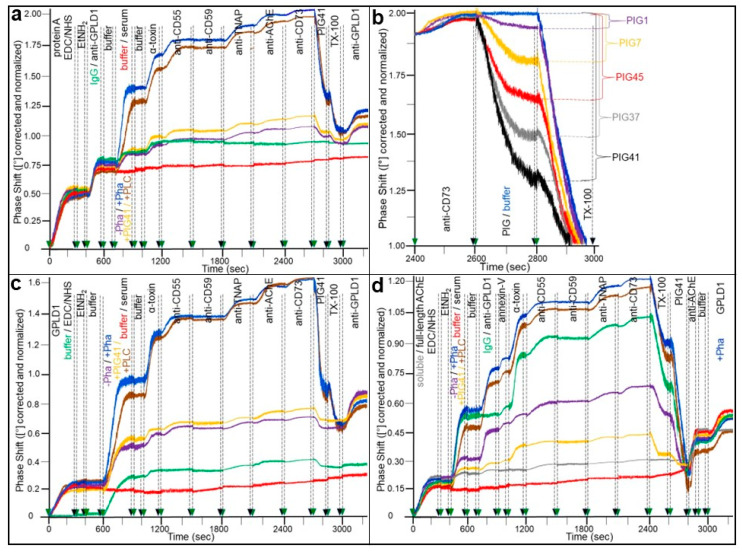
Human serum contains full-length GPI-APs which are bound to and displaced from serum proteins, among them the GPLD1, by PIGs (for structure and design of PIGs, see [146]) (a complete description of the methods and results is given in the Supplementary Materials). (**a**) After covalent immobilization (0–300 s) and subsequent blockade of unreacted carboxyl groups (300–400 s), anti-GPLD1 antibody (blue, yellow, brown, turquoise, red curves) or IgG (green curve) were injected into the chip channels. Following washing of the chips (600–700 s), 200 μL of human serum containing Pha (green curve) or lacking it (turquoise curve), which had been pretreated with bacterial PI-PLC (brown curve) or remained untreated (blue, yellow, turquoise, green curves) or PBS (red curve) were injected (700–900 s) in the absence (turquoise curve) or presence of Pha (all other curves) or PIG41 (yellow curve). After washing of the channels (900–1000 s), α-toxin (1000–1200 s), and subsequently anti-CD55 (1200–1500 s), anti-CD59 (1500–1800 s), anti-TNAP (1800–2100 s), anti-AChE (2100–2400 s) and anti-CD73 (2400–2700 s) antibodies were injected successively. After injection of PIG41 (2700–2850 s) together with Ca^2+^ and then TX-100 (2850–3000 s), anti-GPLD1 antibody was finally injected (3000–3300 s). Phase shift is given upon correction for unspecific interaction of serum components and altered viscosity of the sample fluid. (**b**) The experiment was performed as described (see (**a**); chips with immobilized anti-GPLD1) with human serum in the presence of Pha and subsequent injection of PIGs or buffer together with Ca^2+^ (blue curve). The measured phase shift was corrected (see (**a**)) and is only shown from the start of the last antibody (2400 s) to the end of TX-100 injection (3000 s). (**c**) After covalent immobilization (0–300 s) of human GPLD1 in contrast to buffer-treated chips (green curve), unreacted carboxyl groups were blocked (300–400 s). Following washing of the chips (400–600 s), human serum (blue, yellow, brown, turquoise, green curves) containing Pha (blue curve) or lacking it (turquoise curve) which had been pretreated with bacterial PI-PLC (brown curve) or remained untreated (blue, yellow, turquoise, green curves) or buffer (red curve) were injected (600–900 s) in the absence (turquoise curve) or presence of Pha (all other curves) or PIG41 (yellow curve). After washing of the channels (900–1000 s), α-toxin (1000–1200 s), and subsequently anti-CD55, anti-CD59, anti-TNAP, anti-AChE and anti-CD73 antibodies were injected successively. After injection of PIG41 (2700–2850 s) together with Ca^2+^ and then TX-100 (2850–3000 s), anti-GPLD1 antibody was finally injected (3000–3300 s). The measured phase shift was corrected (see (**a**)). (**d**) After covalent immobilization of full-length AChE or soluble AChE (grey curve) onto chips (0–300 s) and blockade of unreacted carboxyl groups (300–400 s), human serum containing Pha (blue curve) or lacking it (turquoise curve) which had been pretreated with bacterial PI-PLC (brown curve) or remained untreated (blue, yellow, turquoise, green curves) or PBS (red curve) were injected (400–600 s) in the absence (turquoise curve) or presence of Pha (all other curves) or PIG41 (yellow curve). After washing of the channels (600–700 s), monoclonal anti-GPLD1 antibody or IgG (green curve) (700–900 s), human annexin-V (900–1000 s), α-toxin (1000–1200 s), and subsequently anti-CD55 (1200–1500 s), anti-CD59 (1500–1800 s), anti-TNAP (1800–2100 s), and anti-CD73 (2100–2400 s) antibodies were injected successively. After injection of TX-100 (2400–2600 s) and then PIG41 together with Ca^2+^ (2600–2800 s), anti-AChE antibody (2800–2900 s), then buffer (2900–3000 s) and finally GPLD1 (3000–3300 s) together with Pha were injected. The measured phase shift was corrected (see (**a**)).

Finally, binding of full-length GPI-APs to proteins in rat serum was detected, among them to (catalytically inactive) GPLD1 [143]. To test for the possibility that full-length GPI-APs contained in human serum are also bound to proteins and become displaced by PIGs, serum proteins from normal male human probands were assayed for binding of serum full-length GPI-APs and their displacement by synthetic PIGs (Figure 2) (for structure and synthesis of PIGs, see [146]). This experiment was performed as described previously [143], with the only difference being the use of human instead of rat serum. Since GPLD1 has been identified so far as the only serum protein which interacts with full-length GPI-APs [147,148,149,150], the experimental demonstration of this interaction was favored and studied in the presence of ortho-phenanthroline (Pha) which inhibits the Ca^2+^-dependent lipolytic cleavage of substrate GPI-APs by GPLD1 [148,149].

For this, SAW chips were generated with protein A being covalently coupled to the gold surface and a monoclonal antibody cross-reactive for human GPLD1 subsequently being immobilized at their channels (Figure 2a). Both steps (0–400 and 400–700 s respectively) were monitored by considerable increases in phase shift. The injection of serum from normal human probands (700–1000 s) together with Pha (Figure 2a; blue curve), but not without Pha (turquoise curve), caused additional upregulation of phase shift vs. buffer (red curve) in anti-GPLD1 antibody (blue curve), but not anti-IgG control (green curve) channels. Phase shift was further elevated by injection of the GPI-interacting protein α-toxin (1000–1200 s) [151,152], anti-CD55 (1200–1500 s), anti-TNAP (1800–2100 s), and anti-AChE (2100–2400 s), but not of anti-CD59 (1500–1800 s) and hardly of anti-CD73 (2400–2700 s) antibodies, in successive fashion (Figure 2a). This was compatible with binding to rather than cleavage by human serum GPLD1 of GPI-APs in the absence of Ca^2+^, among them CD55, TNAP, and AChE, which all represent minor constituents of human serum.

The roughly 50% decrease in the α-toxin- and antibodies-induced phase shifts following PIG41 and TX-100 injections (Figure 2a; blue curve) argued for the involvement of the GPI anchor glycan core and micelle-like complexes constituted by GPI-APs, cholesterol, and (lyso)phospholipids [142], respectively, in the recognition of GPI-APs by GPLD1. The former was confirmed by co-injection of PIG41 which led to about a 75% reduction in the serum-, α-toxin-, and antibodies-induced phase shift increases (Figure 2a; orange curve). This hinted at a critical role of the GPI glycan core in the interaction of GPLD1 and full-length GPI-APs. Analysis of the potency of structurally different PIGs (for structural details, see [146]) in displacing GPI-APs from human serum GPLD1 revealed that PIG41 was most efficient, followed by PIG37, PIG45, and PIG7, and lastly PIG1, in that ranking order of decreasing potency (Figure 2b).

In contrast to rat serum, pretreatment of human serum with bacterial PI-PLC (700–900 s) led to only a marginal reduction in phase shift increase (Figure 2a; brown curve). This is explained best by the very low deacylation of the *myo*-inositol residue of the GPI anchor of GPI-APs expressed in those human tissues which release full-length GPI-APs into the circulation, since this is the prerequisite for their cleavage by bacterial PI-PLC (see above). The considerable increases in phase shift upon final injection of a polyclonal antibody against human GPLD1 in all channels, except for those with no GPLD1 or serum injected (Figure 2a; green and red curves, respectively), confirmed capture of GPLD1 from the serum of the human probands. Taken together, SAW sensing using chips with immobilized anti-GPLD1 antibodies can be used for analysis of the interaction of full-length GPI-APs and GPLD1 in human serum and their displacement by PIGs and revealed both identities (CD55, TNAP) and differences (CD59, AChE, CD73) in the full-length GPI-APs which are bound to serum GPLD1 as well as in the degree of deacylation of their GPI anchor (high in rats, low in humans).

The capability of human serum GPLD1 to interact with human serum full-length GPI-APs was further validated by the ionic/covalent coupling of recombinant human GPLD1 to the sensor chips, subsequent injection of human male serum under various conditions, and detection of bound serum GPI-APs upon successive injection of α-toxin known to specifically interact with the GPI glycan core, and antibodies against selected GPI-APs and final measurement of the SAW phase shift as has already been performed for (a) (Figure 2c). Successful coupling of the GPLD1 and binding of serum proteins (turquoise curves) was demonstrated by phase shift increases in contrast to corresponding buffer injections (green and red curves, respectively). The presence of Pha during serum injection (Figure 1a; blue curve) led to further phase shift increase. Phase shifts were further and sequentially upregulated by injection of α-toxin, anti-CD55, anti-TNAP, and anti-AChE, but not of anti-CD59 and hardly of anti-CD73 antibodies (Figure 2c). Thus, the types and relative amounts of human serum GPI-APs bound to human recombinant GPLD1 in the absence of Ca^2+^ were identical to those identified in the course of the above experimental configuration with authentic serum GPLD1, captured from human serum together with bound GPI-APs (see Figure 2a).

The drastic decreases of the α-toxin- and antibodies-induced phase shifts following PIG41 and TX-100 injections (Figure 2c; blue curve) confirmed the role of the GPI anchor glycan core and micelle-like complexes constituted by full-length GPI-APs, cholesterol, and (lyso)phospholipids, respectively, in the interaction between GPI-APs and GPLD1. In accordance with this, the co-injection of PIG41 caused considerable reductions of the serum-, α-toxin-, and antibodies-induced phase shift increases (Figure 2c; orange curve).

Finally, the interaction of full-length GPI-APs with serum proteins was studied using the reverse experimental configuration compared to the above (Figure 2a–c), with full-length AChE coupled to the chip surface by ionic/covalent capture and subsequent injection of serum from human male probands (Figure 2d). The latter (Figure 2d; turquoise curve), but not buffer (red curve) led to a considerable phase shift increase compared to captured soluble AChE, prepared by lipolytic cleavage of its anchor-harboring version (Figure 2d; grey curve). Phase shift increased further by successive injection of anti-GPLD1 antibodies (700–900 s), but not IgG (Figure 2d; green curve), the phosphatidylserine-binding protein annexin-V (900–1000 s), the glycan core-binding protein α-toxin (100–1200 s), and anti-CD55 (1200–1500 s), anti-TNAP (1800–2100 s) and, to a minor extent, anti-CD73 (2100–2400 s), but not anti-CD59 (1500–1800 s) antibodies. Pha co-injected together with serum drastically increased phase shift (Figure 2a; blue curve), compatible with inhibition of lipolytic digestion by GPLD1 of bound full-length GPI-APs through complexation of divalent cations. As already observed above (Figure 2a,c), pretreatment of human serum with bacterial PI-PLC (400–600 s, presence of Pha) caused only a minor reduction in phase shift increase (Figure 2d; brown curve), which is presumably due to the persistent acylation of the *myo*-inositol residue of the GPI anchor of released GPI-APs in human serum.

Importantly, the apparent interaction of the complexes consisting of full-length AChE and human serum proteins, among them GPLD1, with phospholipids and human serum GPI-APs, as demonstrated by the annexin-V-, α-toxin-, and antibody-induced phase shift increases, respectively, may be explained best by the binding of human serum proteins exhibiting multiple recognition sites for full-length GPI-APs and therefore not being identical with GPLD1, which displays only a single active site according to present knowledge [149]. Those serum GPI-(AP) binding proteins may form a bridge between captured full-length AChE and micelle-like GPI-AP complexes consisting of (lyso)phospholipids, cholesterol, and full-length GPI-APs released into human serum, among them CD55, TNAP, and CD73. Consequently, this sandwich configuration disintegrated upon subsequent injection of TX-100 with about a 30% reduction in total phase shift increase (Figure 2d; 2400–2600 s) due to the relief of the micelle-like complexes and finally of PIG41, leading to almost complete loss of phase shift (2600–2800 s), caused by the dissociation of GPLD1 from AChE. Its persistent capture by the chip surface including the complete GPI anchor (glycan core) was demonstrated by phase shift upregulations upon injection of a monoclonal anti-AChE antibody (Figure 2d; 2800–3000 s) and then, after removal of PIG41 by extensive washing, of recombinant human GPLD1 together with Pha (2900–3000 s).

Taken together, the strength of the interaction was highest for serum proteins from metabolically deranged rats and serum from obese and diabetic probands. Furthermore, GPI-APs were displaced from the serum proteins by synthetic PIGs, with IC_50_ values being lowest for those of closest structural similarity to the GPI glycan core. Thus, in normal human serum micelle-like complexes consisting of (lyso)phospholipids, cholesterol, and full-length GPI-APs interact with various GPI-binding proteins which recognize their GPI glycan core using a single (GPLD1) or multiple binding sites *per* molecule.

**Figure 3:**
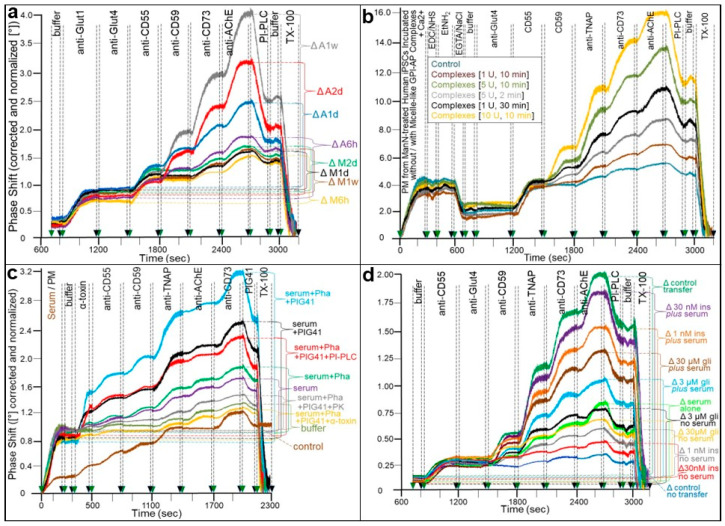
Full-length GPI-APs are transferred from human differentiated cells, human micelle-like complexes, and human serum proteins to human-induced pluripotent stem cells (iPCSs) under the control of PIGs, insulin, and antidiabetic sulfonylureas (the complete legend including the materials used are given in the Supplementary Materials). (**a**) Transfer of full-length GPI-APs from human adipocytes to human iPSCs. (**b**) Transfer of full-length GPI-APs from human micelle-like GPI-AP complexes to iPSCs. (**c**) Transfer to human iPSCs of GPI-APs released from human serum proteins by PIG41. (**d**) Restoration of insulin- and SU-inhibited GPI-AP transfer by serum (a complete description of the methods, results, and their interpretation is given in the Supplementary Materials).

The findings reported so far for human adipocytes and GPI-deficient ELCs as model tissue donor and blood acceptor cells, respectively, prompted the question as to whether transfer of GPI-APs in parallel to a change in the metabolic phenotype is restricted to terminally differentiated somatic cells or also holds true for undifferentiated acceptor cells, such as induced pluripotent stem cells (iPSCs). iPSCs are adult cells that have been reprogrammed to an embryonic stem-cell-like state. These cells can replicate indefinitely in the undifferentiated state (as used here), or differentiate into any other cell types such as nerve, heart, or liver cells under controlled conditions. The EBiSC stem cell bank, from which these iPSCs have been derived, is a collection of human iPSCs which is available for academic and commercial researchers for the use in disease modelling and other forms of stem cell research. The initial collection of cell lines has been generated from a wide range of donors representing specific disease backgrounds as well as from healthy control donors, which have been matched for age and sex. The experiments (Figure 3) were performed with the aid of the transwell co-culture as has been described previously [136,143], with the only difference being the use of iPSCs instead of ELCs as acceptor cells.

For this, differentiated human adipocytes as donor and iPSCs as putative acceptor cells were cultured in the insert wells at the top and in the companion bottom wells, respectively, of transwell co-cultures, which are separated from one another by a semipermeable membrane (with pores of 50 nm diameter) (Figure 3). This configuration prevented direct cell-to-cell contact, but enabled passage of large protein aggregates and complexes such as lipoprotein-like particles (LLPs) and micelle-like GPI-AP complexes, but not of EVs and cells. Upon incubation, the intercellular transfer of GPI-APs, which involves their (i) release from donor cells in the insert wells, (ii) diffusion across the membrane to the bottom wells, and (iii) final insertion into the PMs of acceptor cells, may occur in parallel to a change in the metabolic phenotype. On the basis of dermis fibroblasts representing the primary cells from which the iPS cell line has been derived, stimulation of lipid synthesis was analyzed as the major metabolic phenotype (synthesis of triacylglycerols and phospholipids of the skin) of those putative acceptor cells. Transfer to their PMs was monitored using chip-based SAW biosensing (Figure 3a) as described previously [143], and lipid synthesis was assayed as the incorporation of fluorescent fatty acid (NBD-FA) into total acylglycerols (Supplementary Materials, Figure S2a).

The incubation of human adipocytes as donor cells, but not of medium alone, with human iPSCs, which had been pretreated with mannosamine (ManN) to downregulate the expression of endogenous GPI-APs as previously demonstrated for various differentiated cell lines such as ELCs [136,143], as acceptor cells in transwell co-culture led to considerable increases in phase shift, reflecting the upregulation of PM expression of the GPI-APs CD59, CD73, and AChE, but not CD55, during incubation for 6 h to 1 week (Figure 3a). In contrast, PM expression of the transmembrane proteins, Glut1 and Glut4, did not increase. Interestingly, there were considerable differences in the residual expression of endogenous GPI-APs upon their downregulation by ManN, being high for CD55, moderate for CD73 and AChE, and missing for CD59. Thus, full-length GPI-APs are transferred from micelle-like GPI-AP complexes to human iPSCs with reduced PM expression of GPI-APs, independent of the level of the endogenous GPI-APs, as is reflected in the transfer of both CD55 and CD59 (Figure 3a). Taken together, these findings are compatible with (i) the release of GPI-APs from differentiated human adipocytes in the insert wells, (ii) their subsequent diffusion across the membrane into the bottom wells, and (iii) their final insertion into PMs of undifferentiated human iPSCs with low or missing expression of GPI-APs.

Next, the transfer of exogenous GPI-APs to ManN-treated human iPSCs was investigated by incubation of the latter with increasing amounts of micelle-like GPI-AP complexes, which had been reconstituted from (lyso)phospholipids and total human adipocyte GPI-APs. Upon immobilization of the PMs, prepared from the iPSCs, to the chip and subsequent injection of antibodies, the phase shift increases served as a measure for the transferred transmembrane proteins and GPI-APs (Figure 3b). As expected, the expression level of Glut4 did not differ between cells incubated in the absence (Figure 3b; Control, blue curve) and presence of micelle-like GPI-AP complexes. At variance, incubation with complexes led to considerable increases in expression of each of the human adipocyte GPI-APs CD59, TNAP, CD73, and AChE compared to the control, which were dependent on the amount of complexes and incubation time (Figure 3b). As apparent from the missing expression of CD55 in human adipocytes (Figure 3a), this GPI-AP was not contained in the micelle-like GPI-AP complexes derived from them and therefore failed to be transferred to human iPSCs via those complexes (Figure 3b).

Subsequent injection of bacterial PI-PLC led to about a 30% reduction in phase shift increase. This argued for the GPI anchorage of the proteins at the PMs of the iPSCs and thus for the transfer of at least a considerable portion of full-length GPI-APs (Figure 3b). The complete abrogation of phase shift increases upon injection of TX-100 was compatible with the transfer of the GPI-APs into the PMs of the iPSCs. Taken together, the data demonstrated that human iPSCs with impaired expression of endogenous GPI-APs at PMs can be used for studying the functional effects of the transferred GPI-APs.

The findings that full-length GPI-APs of human serum become displaced from human serum GPLD1 (Figure 2a–c) and presumably other GPI-binding proteins (Figure 2d) by PIGs raised the possibility that GPI-APs are transferred to iPSCs upon their incubation with human serum and PIGs. To test for this, the amounts of total and individual GPI-APs expressed at the PMs, which had been prepared from the intensively washed iPSCs after incubation, were measured by chip-based SAW biosensing with α-toxin and CD55, CD59, TNAP, AChE, and CD73 antibodies.

PMs from ManN-treated iPSCs incubated with serum from healthy probands (Figure 3c; turquoise curve) compared to buffer (Figure 3; olive green curve) expressed considerably elevated amounts of total and individual GPI-APs, as reflected in corresponding successive α-toxin- and CD55, CD59, TNAP, and CD73, but not AChE antibody-induced phase shift increases, respectively. These were further upregulated by the presence of Pha during serum preparation (Figure 3c; dark green curve), PIG41 during serum injection (Figure 3c; black curve) and Pha and PIG41 in combination (Figure 3c; blue curve) in that ranking order of increasing efficacy. This demonstrated the transfer of CD55, CD59, TNAP, and CD73, but not AChE, from human serum GPI-binding proteins to the iPSCs, which was most efficient during (i) inhibition of serum GPLD1 and concomitant stabilization of the interactions between GPI-binding proteins and GPI-APs by Ca^2+^ removal (i.e., presence of Pha) during serum preparation, (ii) destabilization of those interactions by Ca^2+^ (i.e., absence of Pha) during serum injection, and (iii) displacement of the GPI-APs from the GPI-binding proteins by PIG41 during serum injection. Phase shift increases caused by the immobilized PMs did not vary significantly under either condition (Figure 3c; 0–300 s). This was compatible with only subtle mass loading onto the chip due to the transfer of GPI-APs and excluded unspecific binding of serum proteins to the PMs. Unspecific binding of serum proteins to the chip channels was assessed by injection of serum into chips lacking immobilized PMs. It accounted for only about 25% of the α-toxin- and antibody-induced phase shifts (Figure 3c; brown curve). Nevertheless, the accompanying very minor successive phase shift increases confirmed the apparent interaction of serum proteins with GPI-APs in general, and CD55, CD59, TNAP, and CD73, but not AChE, in particular.

The specificity of transfer of GPI-APs was corroborated by drastic decreases in α-toxin- and GPI-APs antibodies-induced phase shifts upon injection of α-toxin Sepharose beads together with the serum (Figure 3c; yellow curve). This presumably interfered with insertion of the GPI anchor into the PMs due to α-toxin binding to the GPI glycan core. Pretreatment of the serum with proteinase K (Figure 3c; grey curve), which degrades the GPI-AP protein moiety, and bacterial PI-PLC (Figure 3c; red curve), which cleaves off the GPI anchor diacylglycerol moiety, led to similar phase shift decreases.

Importantly, injection of PIG41 led to minor reduction of the phase shift increases for each incubation condition (Figure 3c; 1950–2150 s) at an extent comparable to those of the corresponding α-toxin-induced increases (300–500 s). This confirmed the specificity of the recognition of the GPI glycan core by α-toxin. Furthermore, the final injection of TX-100 caused complete loss of the remaining phase shift increases for each incubation condition (Figure 3c; 2150–2300 s), compatible with the insertion of the transferred full-length GPI-APs into the PMs of the ManN-treated iPSCs.

In fact, insulin alone (Figure 3d; no serum, grey and red curves), glimepiride alone (Figure 3d; no serum, black and orange curves), and serum alone (Figure 3d; light green curve) decreased transfer of the GPI-APs studied in a concentration-dependent fashion by up to 90, 80, and 75%, respectively, compared to the efficient control transfer (Figure 3d; dark green curve). The latter became evident from comparison with the absence of donor cells (Figure 3d; control no transfer, dark blue curve). This indicated the efficient reduction of the amount of GPI-APs being competent for transfer, i.e., of those equipped with the full-length GPI anchor in the free, not protein-bound, state in response to insulin, glimepiride, or serum. Importantly, the combination of insulin or glimepiride and serum in the presence of Pha led to concentration-dependent restoration of transfer to about 75–90% (insulin; Figure 3d; light brown and turquoise curves) and 40–65% (glimepiride; Figure 3d; light blue and dark brown curves) compared to maximal transfer in the absence of both (Figure 3d; control transfer).

Previously, two distinct modes of inhibition of the transfer of GPI-APs from donor to differentiated human acceptor cells and concomitantly of transfer-induced alteration of the (metabolic) phenotype in the differentiated acceptor cells have been reported [143]: (i) lipolytic cleavage of the GPI anchor by GPI-PLC in response to insulin and antidiabetic SU drugs of the second and third generation and (ii) interaction of the GPI anchor with serum proteins such as GPLD1 and BSA. On the basis of the above finding of GPI-AP transfer to undifferentiated acceptor cells (Figure 3), its putative inhibition by insulin and SUs as well as the potential sub-additive, additive, or synergistic interactions between the underlying molecular mechanisms were studied using human iPSCs and serum from healthy male probands.

Taken the data together, insulin, the anti-diabetic SU of the third generation glimepiride, and serum were each found to interfere with the transfer of GPI-APs from human adipocytes to human iPSCs with concomitantly reduced GPI-AP expression in a concentration-dependent fashion at physiological and pharmacological concentrations, respectively. However, both the insulin and the glimepiride inhibition was antagonized by serum added to the transwell co-culture. Apparently, the full-length GPI-APs, which were transferred to the iPSCs in the simultaneous presence of serum and insulin or glimepiride, did originate from serum proteins loaded with GPI-APs rather than from the donor human adipocytes. Therefore, this “indirect” mode of transfer of GPI-APs, i.e., from serum proteins with loaded GPI-APs, can be differentiated from the “direct” mode, i.e., from donor cells in the absence of serum proteins or in the presence of serum proteins which are not occupied by GPI-APs. This differentiation corresponds to that which has already been described for the release of GPI-APs from PMs of donor cells and tissues in response to insulin or SUs and their metabolic state, respectively. Importantly, the interaction of human serum GPLD1 and a multitude of human serum full-length GPI-APs has been demonstrated here (see Figure 2). In fact, recent experimental evidence has suggested that GPLD1 and most likely additional serum proteins operate as binding entities, i.e., GPI-binding proteins, for full-length as well as lipolytically cleaved GPI-APs via recognition of the highly conserved GPI glycan core [153,154]. It is tempting to speculate that in the absence of insulin or glimepiride only a minor portion of the binding sites of GPI-binding proteins will be occupied by full-length GPI-APs. Thus, human serum may interfere with or “indirectly” promote transfer which is dependent on the metabolic phenotype of the organism. With regard to the latter mode, GPI-APs which have been lipolytically released from the PMs of donor human adipocytes upon challenge with insulin or glimepiride act as naturally occurring competitors for the displacement of full-length GPI-APs from human serum GPI-binding proteins such as GPLD1, thereby initiating their transfer to human iPSCs.

Importantly, the iPSCs which were analyzed as acceptor cells for the transfer of GPI-APs from human donor adipocytes (Figure 3a,b) as well as its control by various agents, serum, insulin, and glimepiride (Figure 3c,d) have been studied for lipid synthesis to elucidate a putative role of transfer in determining the metabolic phenotype (Supplementary Materials, Figure S2). In fact, a positive correlation between upregulation of transfer of GPI-APs to and lipid synthesis in human iPSCs in course of the simultaneous incubation with serum and insulin or glimepiride was observed. This is compatible with full-length GPI-APs (not identical with TNAP, CD73, and AChE) mediating the upregulation of basal lipid synthesis upon their dissociation from certain serum GPI-binding proteins (not identical with albumin), and their subsequent transfer to the PMs of the iPSCs.

Strikingly, the GPI-APs displaced from rat serum GPI-binding proteins, (i) by excess of PIGs (in the absence of donor cells) or (ii) in the absence of PIGs in response to challenge of donor cells with insulin or glimepiride, were transferred to acceptor ELCs. This resulted in the upregulation of glycogen synthesis with potencies in positive correlation to the concentration of insulin and SUs and to the structural similarity of the PIGs to the GPI glycan core [143]. These findings can best be explained with PIG-proteins, which are generated from full-length GPI-APs of the donor adipocytes in response to insulin or SUs by insulin-/SU-induced GPI-PLC, dissociating the full-length GPI-APs from human serum GPI-binding proteins.

**Figure 4:**
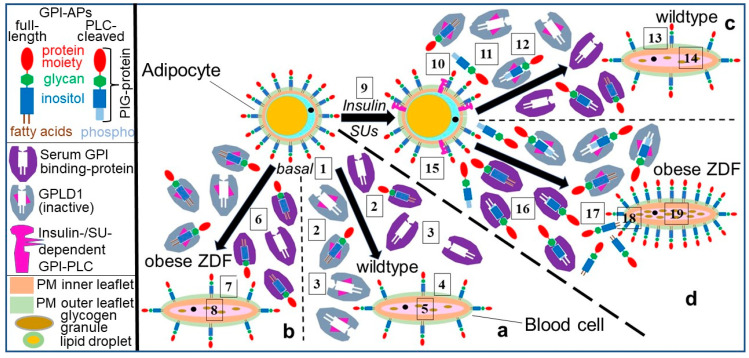
Working model for the “indirect” mode of (“horizontal”) (“downward”) transfer of GPI-APs over a long distance from large lipid-loaded adipocytes to blood (or undifferentiated somatic) cells with the accompanying anabolic effects and its control in the absence (**a**,**b**) and presence of insulin or SUs (**c**,**d**) and serum GPI-binding proteins from normal (**a**,**c**) and metabolically dysregulated rats (**b**,**d**).

In conclusion, both insulin and SUs (of the third generation) manage either to block or to foster transfer when serum GPI-binding proteins are depleted of or loaded with full-length GPI-APs, respectively, i.e., in the normal or metabolically deranged state of rodents and humans. The transfer in vitro of the anabolic state from somatic to blood or stem cells over a long distance and its “indirect” complex control by insulin, SUs, and serum GPI-binding proteins support the (patho)physiological relevance of the intercellular transfer of GPI-APs. It will be of great importance to identify the relevant GPI-binding proteins as well as the bound GPI-APs. To achieve this, the recently described synthesis of bifunctional GPI anchor analogues with conserved glycan core (i.e., photoactivatable diazirine or azido groups at the fatty acyl chains and clickable alkynyl groups at the [glucosamine residue] of the glycan core) may represent valuable probes. With their use, the immediate vicinity of the lipidic anchor portion of GPI-APs can be tested for interacting GPI-binding proteins by photocrosslinking [154,155] (for a review, see [156]). A model for the “indirect” mode of transfer of GPI-APs over a long distance from donor adipocytes via serum GPI-binding proteins to acceptor blood (and undifferentiated somatic) cells with the accompanying upregulation of glycogen synthesis (and lipid synthesis, respectively) and its control by insulin and SUs of the third generation, which is based on previous studies [136,143] as well as on the extension of those data (Figure 2, Figure 3 and Figure S2) is presented (Figure 4).

(Step 1, numbering of the distinct steps as depicted in the figure) Human adipocytes in the basal state (i.e., absence of insulin or antidiabetic SUs) release full-length GPI-APs from the PMs, dependent on their metabolic state and age. (2) The released adipocyte full-length GPI-APs interact with serum GPI-binding proteins (such as catalytically inactive GPLD1 due to the absence of Ca^2+^) from wild-type rats of normal feeding state, which are not loaded with GPI-APs (3), and are thereby prevented from the insertion into the PMs of blood cells (4). The missing GPI-AP transfer is compatible with low basal glycogen synthesis in the blood cells (5). (6) The released adipocyte full-length GPI-APs interact with serum GPI-binding proteins from obese ZDF rats (i.e., of metabolically severely dysregulated state), which are (only) partially loaded with GPI-APs. Consequently, full-length GPI-APs are completely scavenged and thereby prevented from insertion into the PMs of blood cells (7). The missing GPI-AP transfer is compatible with low basal glycogen synthesis in the blood cells (8). (9) Upon exposure to insulin or antidiabetic SUs, insulin-/SU-induced GPI-PLC cleaves the GPI anchor of some GPI-APs at the PMs of human adipocytes (10). The lipolytically cleaved GPI-APs with the GPI anchor remnant, which resembles the structure of PIGs, remain attached (PIG-proteins) and interact with serum GPI-binding proteins from wild-type rats (11), which are not loaded with GPI-APs (12). Consequently, full-length GPI-APs are not made available for the insertion into the PMs of blood cells (13). The missing GPI-AP transfer to blood cells is compatible with low basal glycogen synthesis (14). (15) The lipolytically cleaved adipocyte GPI-APs, i.e., PIG-proteins, generated by the insulin-/SU-induced GPI-PLC (but not inositol-glycan [IG]-proteins, eventually generated by GPLD1 and lacking the terminal phosphate moiety) displace the full-length GPI-APs loaded onto the serum GPI-binding proteins of obese ZDF rats. This depends on the relative concentrations and binding affinities of the PIG-proteins vs. the full-length GPI-APs (16). Full-length GPI-APs displaced from the serum GPI-binding proteins (17) insert into the PMs of blood cells (18). The transferred serum GPI-APs stimulate glycogen synthesis in blood cells with the accompanying accumulation of glycogen granules (19). This is most pronounced at low concentrations of glucose, thus leading to increased sensitivity of the cells towards glucose-driven glycogen synthesis and an elevated level of basal glycogen synthesis. The full-length GPI-AP enroute from human donor adipocytes to acceptor blood cells before, during, and after interaction with the GPI-binding proteins may be arranged in micelle-like complexes together with (lyso)phospholipids and cholesterol, alone or together with other GPI-APs of the same or different type (not depicted in this working model), as have been characterized previously [142].

The efficacy of GPI-AP transfer from donor to acceptor cells and in concert with the induction of anabolic effects such as the upregulation of glycogen granule and lipid droplet biogenesis, as described here and previously [136,143], is determined by the following parameters: (i) the amount of full-length GPI-APs released from donor cells in response to their metabolic state and age, (ii) the concentration of PIG-proteins generated in response to insulin and antidiabetic SUs of the third generation, (iii) the amount and activity of serum GPI-binding proteins, among them GPLD1 (the activity of the latter is controlled by Ca^2+^ and kept inactive by Ca^2+^ chelation in the corresponding experiments, as assumed in this working model), and (iv) the actual load of the total serum GPI-binding proteins with full-length GPI-APs, which depends on the metabolic state and age of the mammalian organism in general, and the donor cells in particular.

In the hyperglycemic and hyperinsulinemic state, the high concentrations of full-length GPI-APs, which had been released from the PMs of donor cells (“direct” mode) or displaced from serum GPI-binding proteins by PIG-proteins (“indirect” mode) and still remain or subsequently become assembled into micelle-like complexes, may be reduced by the action of serum GPLD1. Interestingly, GPLD1 activity has recently been shown to become upregulated in the course of the development of insulin resistance, metabolic syndrome, obesity, and type 2 diabetes [142], and by age [134]. Considering this complex interplay of serum GPI-binding proteins, insulin, and antidiabetic SUs of the third generation, it seems difficult to predict whether the transfer of full-length GPI-APs with its accompanying anabolic effects or their degradation by serum GPLD1 will dominate during metabolic derangement and ageing. Moreover, additional and so far unknown (serum) factors may affect the steady-state between transfer and degradation of full-length GPI-APs in response to certain endogenous and environmental cues, thereby enabling the fine-tuning of intercellular transfer and anabolic effects.

It seems likely that additional (patho)physiological phenomena elicited by the “indirect” mode of intercellular transfer of GPI-APs will be discovered in the near future. In this regard, the previous findings that the serum concentrations of GPLD1, which degrades GPI-APs after their release into the circulation of mammals, including humans, are elevated in aged mice in response to exercise in correlation to improvement of their cognitive function as well as in healthy active elderly human probands [157], already hint at an additional candidate mechanism. Importantly, the injection of GPLD1 into aged mice led to the amelioration of age-dependent regenerative impairment. This has been interpreted as a result of the induction of signaling cascades downstream of GPI-APs in the course of their lipolytic cleavage in the blood and subsequent transport into the brain [157]. Alternatively, it is conceivable that the IG-proteins, i.e., GPI-APs cleaved by the exercise-induced GPLD1, act as competitors for the full-length GPI-APs, which are loaded onto the serum GPI-binding proteins, as has been demonstrated for PIG-proteins generated by the insulin-/SU-induced GPI-PLC. Upon displacement from the GPI-binding proteins, the full-length GPI-APs could be transferred to the PMs of acceptor cells in the brain, which would result in beneficial effects on neurogenesis and cognition in aged mice and humans.

During the past three decades, the transfer of full-length GPI-APs (recombinant fusion protein-GPI) from vesicles (microvesicles, exosomes, liposomes reconstituted with GPI-APs) or non-vesicular structures (LLPs, aggregates, micelle-like complexes) to the surface of acceptor cells or other biomolecular surfaces (e.g., isolated PMs, enveloped viruses, albumin microparticles), often called “cell surface painting“, “membrane painting“, “surface engineering“, or “protein paints” in the literature, has been successfully used for numerous biomedical and biotechnological applications. These include: 37-kDa merozoite surface protein for vaccination against babesiosis [158]; immunostimulatory factors, such as granulocyte-macrophage colony-stimulating factor, for vaccination against heterologous influenza strains [158]; co-immunostimulatory proteins CD80 and ICAM-1 to albumin microparticles as antigen delivery devices for vaccination and targeted drug delivery [159,160]; CD80 (B7-1), interleukin-12 (IL-12), and HER-2 for human immunotherapy and vaccination to induce potent antigen-specific immunity and tumor regression (here breast cancer) or to prevent postsurgical secondary metastases [161,162,163,164]; CD80 expressed in recombinant baculovirus to human tumor cells in functional form for immunotherapy [165]; CD80 and IL-12 to tumor PMs for vaccination against head and neck squamous cell carcinoma and personalized immunotherapy that enhances the efficacy of immune checkpoint inhibitors [166]; CD80 and CD86 to murine tumor cell lines under maintenance of their immune costimulatory function for vaccination against cancer [167]; breast cancer antigen HER-2 to virus-like particles for vaccination against cancer [168]; human IL-12 to tumor PMs as potent immunogenic tumor vaccines [169]; HLA-A2.1 for the presentation of antigen (here hepatitis B virus peptide) complexes to cell surfaces [170]; folate receptors for targeted drug (e.g., imaging agents, chemotherapeutic agents, nanoparticles) delivery for chronic inflammatory diseases and cancer [171]; SDF-1 fused to a fractalkine stalk and a GPI anchor for the induction of functional neovascularization for the treatment of chronic ischemic syndromes [172]; CD55 and CD59 for the correction of PNH [173,174]; and GPI-APs displayed on the surface of exosomes as diagnostic markers and drug delivery systems [175,176,177,178,179,180].

**Figure 5:**
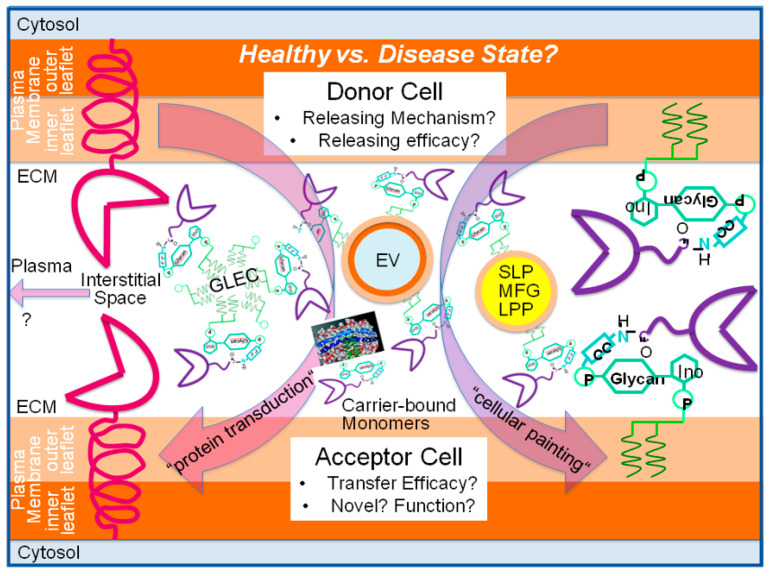
Summary model for the distinct modes of the intercellular transfer of GPI-APs. GPI-APs with the complete anchor located at the outer leaflet of the PM bilayer of tissue or blood donor cells are released into the extracellular matrix (ECM) or interstitial space. From there they can be transferred to the surface of neighboring tissue or blood acceptor cells, either directly within the same tissue bed or via the circulation (resulting in transient appearance in the plasma) following transport across the vascular endothelial cells. Following insertion of the GPI-APs into the outer leaflet of the PM bilayer, which is mediated by their GPI anchor, they may either maintain their original or acquire a novel function to be fulfilled at the acceptor cells. The molecular mechanisms and structural vehicles involved in the release from the donor PMs and passage across the aqueous milieu of the interstitial space/plasma (aggregated in lipid-free homo-multimers, interacting as monomers with GPI-binding or carrier proteins or incorporated into lipid-containing SLPs, LLPs, MFGs, micelle-like GPI-AP complexes or “GLEC” and EVs) as well as in the insertion into the acceptor PMs and the relevance of the transfer event for (patho)physiological outcomes, such as the development of stress-related diseases (e.g., type 2 diabetes, obesity), remain to be determined for each transferred GPI-AP and each mechanism. Furthermore, it cannot be excluded at present that the transfer of GPI-APs may also happen upon the direct and intimate physical contact of the PMs of donor and acceptor cells. This would make the generation of hydrophilic structural vehicles for the GPI-APs unnecessary, which manage to prevent their full-length GPI anchor from access of the aqueous milieu of extracellular compartments. Such a mechanism, so-called trogocytosis, has already been demonstrated to be involved in the transfer of canonical transmembrane proteins from donor to acceptor cells [181,182] (see Section 5).

In conclusion, there is a large body of evidence for the operation of the intercellular transfer of GPI-APs by the engagement of a variety of distinct molecular mechanisms, encompassing both vesicular (exosomes, microvesicles, LLPs) and non-vesicular structures (lipid-free aggregates, GPI-binding or carrier proteins, lipid-containing micelle-like complexes), which can be summarized as follows (Figure 5).

## 5. Some Thoughts about the Evolution of GPI-AP Transfer

In the yeast *Saccharomyces cerevisiae*, a considerable portion of its GPI-APs (around 60) become transferred and coupled to the cell wall and consequently is engaged in cell wall integrity and assembly [183,184], among them Cwp2p [185] and Tip1p [186]. At variance, other yeast GPI-APs remain anchored at the PMs [183] or reside at both locations, among them Gas1p [187] and Gce1p [188]. During the transfer of GPI-APs to the cell wall, the glucosamine moiety of the glycan core together with the PI building block is eliminated and concomitantly the protein moiety becomes coupled to the ß-1,6-glucan of the cell wall via the three mannose residues remaining left at the glycan core [189] in the course of a transglucosylation reaction, which involves Cwg6/GPI-3 [190]. Interestingly, the decision between targeting to the cell wall or the PMs critically depends on the amino acid sequence upstream of the GPI attachment (ω) site [191]. Different (positive or negative) signals for cell wall targeting [192,193] or targeting by default [194] have been proposed so far. Importantly, more recent studies have shown that the type of inositolphosphorylceramide in the GPI lipid portion is involved in the retention of the GPI-APs at the PMs [192,195]. Finally, Cwh43p and the genetically related Ted1p, which encode proteins engaged in the elimination of EtN-P from the second mannose residue of the glycan core [192], as well as Dcw1 and Dfg5p, putative mannosidases [193], may operate as components of a sorting machinery which is localized at or near the PMs and functions by recognizing the amino acid sequence upstream of the ω-site for the differential transfer of a specific class of GPI-APs from the PMs to the cell wall.

On the basis of the apparent dual localization of GPI-APs in yeast with some of them finally residing at the PMs in the course of trafficking along the typical secretory pathway [195], and others continuing their journey—following their release from the PMs—across the periplasmic space to the cell wall and finally being covalently coupled to its ß-glucan by transglucosylation, it is tempting to speculate about the evolution of the anchorage of membrane proteins by GPI. GPI-APs may have been introduced by yeast operating at both the PMs and the cell surface or cell wall. It is conceivable that, for transglucosylation to occur, the full-length GPI-APs, equipped with the complete glycan core, have to be presented in micelle-like (lyso)phospholipid-containing GPI-AP complexes or EVs to the cell wall ß-glucan and the associated enzymic apparatus. The latter manages to transfer the GPI-APs-ß-glucan intermediates from the PMs across the periplasmic space to the cell wall. Alternatively, protrusions of the PMs, which harbor the envisaged cell wall GPI-APs and come into close contact to those sites of the cell wall, may be involved in the expansion and growth of the cell wall.

The expression of micelle-like GPI-AP complexes or EVs in yeast has not been studied so far. However, previously soluble versions of a subset of full-length GPI-APs, among them Gce1, from the PMs into the periplasmic space of *Saccharomyces cerevisiae* under conditions of glucose repression have been reported [188]. This may be compatible with the signal-induced transfer of full-length GPI-APs with the final destination cell wall in micelle-like complexes or EVs across the aqueous periplasmic milieu, prior to their transglucosylation to the ß-glucan. With the absence of cell walls in higher eukaryotic cells such as in mammalian organisms, the mechanism of the—spontaneous or signal-induced—release of full-length GPI-APs into micelle-like complexes or EVs could have acquired new functions. GPI-APs may succeed in the “direct”, “vertical”, and/or “horizontal” transfer between neighboring tissue cells with accompanying (patho)physiological consequences.

Taken together, it is concluded that GPI-APs may not have lost their functional diversity during evolution but rather have replaced one major role, their involvement in the biogenesis of and protein anchorage at the cell wall of unicellular fungi, with another one of no less importance: GPI-APs enable the intercellular transfer of proteins in multicellular eukaryotic organisms. Nevertheless, this apparent switch in function does not necessarily exclude the—seemingly remote—possibility that in fungi full-length GPI-APs in micelle-like complexes, which have been released into the periplasmic space, manage to pass the (porous network of the) cell wall without transglucosylation. Thereafter, those may reach the environmental medium and undergo transfer to the PMs of neighboring cells, with the accompanying effects on the phenotype of the latter. This transfer of GPI-APs could be interpreted as exchange of information, i.e., communication or of matter (see Section 7) or as non-genetic inheritance (see Section 8) between fungi.

## 6. Some Thoughts about PIGs, Mediators of Insulin Action, and GPI-AP Transfer

As discussed above, anabolic effects, i.e., stimulation of glycogen and lipid synthesis, are elicited in acceptor cells such as human adipocytes or ELCs upon transfer of full-length GPI-APs from donor cells with the concerted action of lipid-containing micelle-like GPI-AP complexes and lipolytically cleaved GPI-APs (PIG-proteins), which are produced in response to physiological stimuli such as hormones (e.g., insulin) or therapeutic agents such as antidiabetic SUs of the third generation (e.g., glimepiride). This raises the question about the relationship of the molecular mechanism underlying this insulin-mimetic activity and the insulin-mimetic activity exerted by the so-called soluble mediators of insulin action (PIGs). The latter were identified almost four decades ago in the incubation media of a multitude of insulin target cells, such as adipocytes, but have resisted purification to homogeneity and unambiguous structural elucidation so far. The previous model of their generation encompassed the two-fold—lipolytic and proteolytic—cleavage within the GPI anchor and at the carboxy-terminus of the protein moiety, respectively, of GPI-APs and, as the underlying molecular mechanisms of their insulin-mimetic action, their specific transport across the target cell PMs and allosteric regulation of the activity of key metabolic enzymes in the cytoplasm in insulin-like fashion (e.g., [196,197,198]; for a review, see [199,200,201,202]). However, this model has remained a matter of intense dispute for decades, as held true for the corresponding data basis [203].

The very recent findings, as summarized above, strongly argue that the insulin-independent stimulation of glucose and lipid metabolism by PIG-proteins, generated in response to physiological and pharmacological stimuli, as well as by synthetic PIGs, added to the incubation medium, in concert with full-length GPI-APs, which interact with GPI-binding proteins, relies on extracellular sites and mechanisms of action. Thus, almost 40 years of research have finally led to a shift in the thinking about the mode of the insulin-mimetic action of PIGs from intracellular to extracellular. This shift coincides with (i) the dissociation of full-length GPI-APs from GPI-binding proteins into the incubation medium of insulin target cells in vitro and the circulation or interstitial spaces of treated rodents in vivo, respectively, and (ii) the subsequent transfer to and insertion into the outer leaflet of the PMs of insulin target cells of the full-length GPI-APs. This extracellular mode of action of PIGs which—by nature—is susceptible towards a multitude of exogenous—experimental, (patho)physiological and environmental—factors may explain the prominent problems with the (high) statistical variance and, in part, even (limited until missing) reproducibility of some of the published findings regarding the insulin-mimetic activity in vitro and in vivo of both isolated and synthetic PIGs (this issue has been discussed extensively in ref. [143], Supplementary Materials).

Furthermore, the extracellular mode of action of PIGs may pave the path for the development of novel anti-diabetic agents. Previous efforts to use the structure of PIGs for the design of insulin-mimetic small molecules with oral bioavailability were severely hampered by the apparently erroneous assumption of the need for cell permeability. Furthermore, it turned out to become difficult to convert the PIG glycan core into a non-carbohydrate structure in order to gain stability during and to increase the efficacy of the gastrointestinal passage for oral absorption of putative insulin-mimetic drugs, which rely on this mode of action. In fact, systematic variation of the individual carbohydrate components of the PIG glycan core as well as of their glycosidic linkages on the basis of experimentally established structure-activity relationship led to the design of PIGs which exerted almost full insulin activity in vitro (e.g., stimulation of glucose transport in primary rat adipocytes, see [146]). However, those efforts failed in the generation of non-carbohydrate PIG-mimetics with insulin-mimetic activity comparable to that of the authentic PIGs as well as in the reduction of the size of the latter in order to reduce the expenditure of their chemical synthesis.

The elucidation of the extracellular site of action of the PIG-proteins which involves the displacement of full-length GPI-APs from serum GPI-binding proteins, among them—but not restricted to—albumin, and the parallel introduction of an innovative chip-based and microfluidic SAW biosensor for its assaying (even in the high-throughput mode) may create novel opportunities of drug discovery research for the pharmaceutical industry, which is engaged in the therapy of metabolic diseases, especially type 2 diabetes and obesity. In particular, in the future efforts to identify serum GPI-binding proteins (different from albumin) could lead to the characterization of those which are equipped with an allosteric site of regulation. It may be of relevance that the activity of GPLD1 has already been demonstrated to be positively controlled by divalent cations [147,148,149]. The use of allosterically controlled GPI-binding proteins in screening efforts may lead to the design of non-carbohydrate small-molecule insulin-mimetic drugs of lower EC_50_ compared to those which rely on the competitive displacement of full-length GPI-APs.

## 7. Transfer of Matter rather than Information

The intercellular transfer of full-length GPI-APs, irrespective of whether they are arranged in EVs such as microvesicles and exosomes or in non-vesicular structures such as LLPs, aggregates, or micelle-like complexes from donor cells (e.g., large primary rat or cultured human adipocytes) or from serum GPI-binding proteins (e.g., of metabolically dysregulated rats or diabetic patients) to acceptor cells (e.g., small primary rat adipocytes, human erythrocytes, or cultured ELCs), with accompanying anabolic effects in the latter (upregulation of lipid and glycogen synthesis, respectively), has to be regarded as a transfer of matter rather than information. This matter is embodied in the transmembrane and soluble proteins, among them a variety of enzymes, receptors, transporters, and structural and adhesion proteins, which become transferred by EVs and non-vesicular structures, as has been amply documented for a multitude of cell types under various (patho)physiological conditions during the past four decades.

At variance, information is encoded in hormones, neurotransmitters, signaling proteins, or nucleic acids. This crucial and often neglected differentiation also holds true for the ongoing canonical assumption of EVs operating as information carriers for intercellular communication by the majority of the research groups active in this area (e.g., [204]; for a review, see [205,206,207,208,209,210,211,212,213,214,215,216,217]). It is based on a misunderstanding of the metaphor “information”, as it is typically used for the realization of “messages” encoded by nucleic acids as well as for the signaling of “messages” triggered by hormones and neurotransmitters. The EVs, aggregates, LLPs, and micelle-like complexes which transfer membrane proteins, including GPI-APs, are built up by discrete biological matter such as enzymes, receptors, structural proteins, transporters, and ion channels which are capable of exerting catalytic, binding, structural, transporting, and conductive functions by themselves. They do not represent an abstract message, signal, or order encoding one of these functions, which has to be deciphered or decoded by a sophisticated cellular apparatus (e.g., protein translation, signal transduction cascades). This apparent dualism of matter and information is reflected best in the obvious missing relationship between structure and function for information and signaling molecules such as, e.g., the three-dimensional conformation of insulin and the molecular mechanism of glucose transport stimulation (which is the signal encoded by insulin in mammalian cells). In contrast, membrane proteins, including GPI-APs, as transferred by EVs, aggregates, LLPs, and non-vesicular structures, are characterized by clear-cut structure–activity relationships such as glycerol-3-phosphate acyltransferase and lipid synthesis or CD59 and binding to complement factors. Consequently, the discrimination between transfer of matter, as is manifested in the transfer of GPI-APs from donor to acceptor cells, irrespective of the molecular mechanisms and vehicles involved, and transfer of information, as is manifested in the secretion of messenger or signaling molecules from donor cells and their decoding by acceptor cells, should be acknowledged.

For instance, the matter of full-length GPI-APs in EVs and micelle-like complexes is released from large lipid-loaded adipocytes (and possibly glycogen-loaded blood and somatic cells) with higher efficacy compared to small adipocytes (and possibly blood and somatic cells). Conversely, this matter is transferred to small adipocytes (and possibly blood and somatic cells) with higher efficacy compared to large adipocytes (and possibly blood and somatic cells). Thus, transfer of the matter (of full-length GPI-APs) from somatic donor to acceptor cells leads to the gradual acquisition of the “lipid-loaded” anabolic phenotype of the mother cells by the daughter cells, in the course of increasing lipid synthesis, lipid droplet formation, and cell size. It can therefore be regarded as the transfer of (non-genetic) matter (see Section 8), which may be critically affected by the environment surrounding the cells and organisms. A number of factors, among them age, mechanical forces, hormones, drugs, nutrition, and metabolic state, manage to gain direct access to the (site of the) source of the GPI-APs which may be potentially transferred (i.e., the PMs of donor cells, serum GPI-binding proteins) and the nature (i.e., structure, composition, size) of the matter (e.g., “membrane landscape”) to be potentially transferred.

Interestingly, intercellular protein transfer via non-vesicular structures is apparently not restricted to GPI-APs but has also been discovered for the multiple-membrane-spanning erythrocyte anion transporter Band 3 almost four decades ago [84,85,86]. Band 3 becomes transferred from human erythrocytes into the bilayer of sonicated liposomes in the functional state and native configuration, albeit at a considerably lower efficacy compared to GPI-APs [84,85,86,218]. Very recently, the phenomenon of the transfer of transmembrane proteins from exogenous sources to erythrocytes has been re-visited [219]. Fluorescently labeled erythrocytes from knockout mice for the erythrocyte transient receptor potential channel of canonical subfamily member 6 (TRPC6) were transfused into differently labeled wild-type mice. This led to the restoration of the TRPC6 channel function in the KO mice within 10 days, strongly arguing for the intererythrocyte transfer of TRPC6. Importantly, with the aid of a cantilever of an atomic force microscope for mimicking the mechanical challenge and accompanying confocal microscopic imaging, strong interactions with the accompanying generation of tethers between human erythrocytes were detected [219]. Thus, the mechanically stimulated intererythrocyte transfer of transmembrane proteins seems to be mediated by direct cell-to-cell contact, the so-called trogocytosis [220,221,222]. This involves neither vesicular nor non-vesicular mechanisms and vehicles as has been described here so far.

The energy-requiring process of trogocytosis enables an acceptor cell to receive a fraction of matter from a donor cell which is in the immediate vicinity of the former. Thus, the PMs of the donor and acceptor cells are in direct contact. Trogocytosis, which should not be mixed up with phagocytosis, which involves the engulfment of the total cell body, has initially been described for various types of immune cells such as natural killer cells, basophils, neutrophils, macrophages, B cells, T cells, dendritic cells, and innate lymphoid cells (for a review, see [223]), and has subsequently been found to operate also in non-immune cells, among them mesenchymal stromal cells, microglia, and lymph node stromal cells [224,225,226]. During the process of trogocytosis, the acceptor cell receives areas of the PMs from the donor cell, encompassing membrane proteins, membrane phospholipids, and the underlying cytoskeletal elements, i.e., eventually complete “membrane landscapes”. Importantly, it was estimated that about 3% of the membrane proteins of donor natural killer cells become rapidly transferred to acceptor natural killer cells in the course of trogocytosis [227]. In addition to this trogocytosis-mediated transfer of matter, trogocytosis may mediate the death of the donor cell (so-called “trogoptosis”; for a review, see [228]) which, however, is not of relevance for the topic of this review. It is well-known that trogocytosis manages to affect the (patho)physiology of both donor and acceptor cells, i.e., the acceptor cells may gain new functions by acquiring “membrane landscapes” from the donor cells, while the donor cells may lose certain functions due to depletion of those “membrane landscapes” and eventually die. Immune-modulatory effects belong to the best characterized consequences of trogocytosis [221]. Thus, there are good reasons to assume that GPI-APs incorporated into “membrane landscapes” can also be transferred from donor to acceptor cells by trogocytosis in concert with transmembrane proteins. However, so far there are only a few studies reporting the trogocytosis of GPI-APs, among them NKG2D-L between natural killer cells [81] and PrP^C^ between neurons [132].

In conclusion, while trogocytosis may play a considerable role in the intercellular transfer of GPI-APs without the involvement of vesicular structures for specific cell types such as immune cells under specific (patho)physiological conditions such as immune response, direct cell-to-cell contact does represent only one—and presumably a minor one—of several possible non-vesicular mechanisms underlying this phenomenon. This is exemplified by the operation of intracellular GPI-AP transfer in the transwell co-culture with the donor and acceptor cells separated by a membrane, which is permeable for micelle-like complexes and protein aggregates (but not for vesicles, LLPs, and cells) and, importantly, is not compatible with the formation of cell-to-cell contacts [136,143] (Supplementary Materials, Figures S1 and S2). Nevertheless, the findings with TRPC6 [219] apparently broaden the concept of the transfer of (non-genetic) matter and its control by environmental cues to canonical transmembrane proteins. However, on the basis of efficacy and the need for direct cell-to-cell contact, transmembrane proteins as the matter of transfer are likely of lower (patho)physiological relevance compared to GPI-APs.

## 8. Non-Genetic Inheritance of Acquired Features

Today, the transfer of DNA from the daughter to the mother cells and from parents to offspring became, in the mind of most biologists but also lay people and the general public, the only process responsible for the like-begets-like phenomenon. The DNA-centric theory of inheritance represents yet another version of the “donation/conception” theory for the explanation of the like-begets-like phenomenon. This theory has evolved through the ages with corresponding changes in the nature of the matter being “donated” or transferred from the pre-microscope versions of Hippocrates, Aristotle, and others to the gametocentric versions of the 18th century, to the nucleocentric versions of the 19th century, to the chromosomecentric versions of the first half of the 20th century, to the DNA-centric versions of the second half of the 20th century. The assumed nature of the transferred matter has always driven and shaped the process of scientific discovery and has led to many important discoveries. Despite this, the assumption of a unique type of transferred matter in all these versions of the “donation/conception” theory need to be questioned. The “donation/conception” theory in its general form is neutral with respect to the nature of the matter that, by being transferred from mother to daughter cells or parents to offspring at “conception” and being accepted by daughter cells or offspring, explains the like-begets-like phenomenon. In its general form, it only says that such matter exists (for details of the history of the concept of the non-genetic inheritance of acquired features, see Supplementary Materials, Ad 8).

In fact, this transfer of matter that happens at “conception” involves more than genetic material, more than DNA. Today it is known to involve proteins, cytoplasm, RNA compounds of various types, and organelles as well as total and complex “membrane landscapes” such as lipid rafts and specific “protuberances” of the PMs. In this regard, the renewed interest in the structural and functional complexity of so-called “membrane landscapes” may be of considerable relevance [229,230,231,232]. All organisms start their life not as DNA molecules, but as cells equipped with proteins, RNAs, cytoplasm, organelles, and “membrane landscapes”. Additionally, in virtue of their parentally derived non-DNA cellular constituents, organisms have many non-genetic “conceptional” features. An example could be the feature of having a given RNA compound in a given section of the cytoplasm of the zygote, thereby forming gradients of developmentally relevant matter. Another example would be the feature of having a specific protuberance at a given PM domain or lipid raft of the daughter cell, thereby forming a template or matrix for its self-organization and thereby replication. Importantly in this regard, self-organization must not be mixed up with the self-assembly of biological structures from pre-formed components, exclusively, without the need for any entity that acts as a template or matrix and is not contained in the final structure, which only holds true for structures free of (phospho)lipids and membranes such as ribonucleoprotein particles (e.g., ribosomes) and bacteriophages (e.g., T2, T4). At variance, cells and animal viruses have to transfer their organelles and “membrane landscapes” of specific orientation and topology to their “offspring” to enable the self-organization of their matter. Thus, the inheritance of biological features should be studied using different perspectives or “agential separability” (see Section 9), with regard to the transfer of genetic and non-genetic materials or of information and matter.

If it is true that the transfer of information or matter is sufficient to explain the reliable reoccurrence of phenotypes within lineages, then it must be the case that intrinsic conceptional features are sufficient to explain the development of these phenotypes. Richard Lewontin [233,234] emphasized the interesting view that the use of the word ‘development’ to talk about the biological process of living development is metaphorical. ‘Development’ means ‘unfolding’ of something being aggregated but already present. The use of the word ‘development’ to talk about the causal process that goes from the intrinsic conceptional features of an organism to its phenotypes—from the mother to the daughter cell, from the zygote to the adult—somehow suggests that this process is similar to an unfolding: Phenotypes are seen as resulting from an unfolding of what is already present in the daughter cell or zygote. Additionally, this is akin to saying that the daughter cell or the zygote at a very early stage of life is seen as being explanatorily sufficient for the emergence of each of their phenotypes during all later stages of life.

All versions of the “donation/conception” theory are committed to a version of the unfolding theory of development. However, different versions of the “donation/conception” theory are committed to different versions of the unfolding theory. The DNA-centric theory is committed to the idea that inherited phenotypic features can be seen as unfolding from genetic features. In the preformationist version of the 17th century, inherited phenotypes were thought to be transferred as already formed features by mother cells or parents to daughter cells and offspring, respectively, at the moment of “conception”, and the “unfolding” of phenotypes was nothing but the process of a growth in size—without any change in their structure and function. This strongly resembles the biogenesis of eukaryotic organelles and “membrane landscapes” as that proceeds by the incorporation of newly synthesized constituents into pre-existing membraneous structures which act as matrix or template for the correct assembly, i.e., ‘replication’, of organelles and “membrane landscapes”.

The metaphor of development is an attractive metaphor because the unfolding theory is an attractive theory. The unfolding theory is attractive since it is simple in that it explains development without mentioning factors external to the organism, i.e., it does not appeal to features acquired during or after conception. Additionally, it is an attractive theory because it fits very well with the—possibly universal—pre-theoretic intuition according to which living organisms are relatively closed systems: What happens to living organisms is intuitively seen as due in most cases to forces internal to the organisms. Additionally, this applies especially to what happens to organisms during development. However, despite its appeal, the unfolding theory does not seem to be compatible with what still must be learned about the development of phenotypes. In this regard, it seems to be justified to interpret the transfer of GPI-APs via micelle-like complexes and of total complex “membrane landscapes” such as lipid rafts and “protuberances”, harboring GPI-APs via EVs from (differentiated) donor cells such as human adipocytes to (undifferentiated) acceptor cells such as iPSCs, as has already been demonstrated (see Figure 3), or from parents to offspring, which remains to be demonstrated, as molecular mechanisms for the inheritance of acquired features which relies on non-genetic matter.

As can be deduced from above (see Section 7), the matter of biological inheritance is defined as a material or substance which is (i) replicated and (ii) transferred from donor to acceptor organisms, i.e., from mother to daughter cells or parents to offspring, and (iii) causatively or explanatorily sufficient (see below) to explain the reliable (re)occurrence of biological features. For historical reasons, in particular the seminal experiments of bacterial transformation performed by Frederick Griffith almost one century ago [235] and other—still obscure—reasons, DNS has been attributed the sole and unique role of a matter of inheritance. However, for the moment as a theoretical construct and thought experiment, the sequence of events which happens during the acquisition of the feature pathogenicity by the acceptor bacteria during experimental transformation is modified as follows:

(i) The pathogenic—donor—bacteria co-cultured with the non-pathogenic—acceptor—bacteria release certain membrane proteins (rather than DNS fragments encoding them), which confer the pathogenic surface characteristics from the PMs into the culture medium via vesicular or non-vesicular vehicles and mechanisms (rather than cell lysis).
(ii) Those pathogenic membrane proteins (rather than DNS fragments) become transferred to the acceptor bacteria, which lack them.
(iii) Upon incorporation of the transferred pathogenic membrane proteins into specific pre-existing “membrane landscapes” of the PMs, which operate as a matrix or template, and their correct and functional incorporation and assembly into those structures (analogous to the one DNA strand of the double helix acting as a template for the newly synthesized one), the acceptor bacteria will display the typical pathogenic phenotype as exerted by the donor bacteria.
(iv) The pathogenic phenotype will be transmitted from the bacteria with acquired pathogenicity as new donors to the next non-pathogenic acceptor cells in a reliable, stable, and recurring fashion.

In eukaryotic cells, the pathogenic membrane proteins incorporated into the “membrane landscapes” of the above example may be exemplified by “protuberances” and lipid rafts of the PMs harboring GPI-APs, but also by intracellular organelles such as mitochondria and endoplasmic reticulum (ER). This raises the question as to whether “membrane landscapes” fulfill the criteria of a matter of biological inheritance, as defined above, albeit not based on DNA, and consequently can be regarded as a non-genetic (rather than genetic) matter of inheritance. Representatives of the DNA-centric view of inheritance, and those represent the vast majority of modern molecular biologists and geneticists, would certainly argue against the possibility of non-genetic inheritance. This view is predominantly based on the strict dependence on newly synthesized proteins and, in consequence, on DNA replication and transmission of the mechanism copying the “membrane landscapes”.

Undoubtedly, non-genetic inheritance only operates in concert with genetic inheritance. On the other hand, genetic inheritance critically depends on the operation of proteins such as DNA polymerase and “membrane landscapes” such as mitochondria and ER (e.g., providing DNA attachment sites in course of cell division), which become replicated by a “disperse” mechanism of growth (rather than a semi-conservative mechanism of copying). This non-genetic matter is then transferred via vesicular and non-vesicular vehicles and mechanisms from mother to daughter cells in the course of cell division and from gametes to zygotes upon cell fusion.

From this mechanism it should become clear that a lifetime of the individual GPI-AP beyond the lifespan of the cell (irrespective of whether it is the mother or daughter cell, gamete or zygote) does not compromise the inheritance of the matter of “membrane landscapes” harboring specific GPI-APs such as lipid rafts and PM “protuberances”. The transferred “membrane landscapes”, which are specifically derived from the mother cells or gametes, will operate in the daughter cells and zygotes, respectively, as “matrix, template or skeleton” for the incorporation of newly synthesized constituents, among them the corresponding GPI-APs. Therefore, those will obtain the correct configuration, arrangement, or assembly state within the specific “membrane landscape”. Consequently, the non-genetic material does not encode the information required for synthesis of the constituents of the “membrane landscapes”, as is the case with DNA. Rather, the non-genetic material can be interpreted as the *intra-action* (see Section 9) between information and matter for the biogenesis, i.e., replication, of “membrane landscapes” (including their environmentally induced alterations and adaptations) in a structural and functional correct configuration.

Nevertheless, the non-genetic inheritance of matter may be interpreted as explanatory background only, which does not deserve to be mentioned explicitly for an explanation of a like-beget-like phenomenon. According to this widely accepted view, DNA as the matter of biological inheritance in the foreground is explanatorily, but—admittedly—not causally sufficient. Thus, the DNA-centric view could be acceptable unless it would not detract from or even deny the possibility of the inheritance of biological features which have been acquired during the lifespan of the organism.

Certainly, and in accordance with the central dogma of molecular biology [236], there is no path from extrinsic (e.g., environmental) factors to the DNS as well as from somatic cells which were exposed to those factors to germ line cells, leading to a (partial) re-writing of the individual “book of life” [237]. Conversely, “membrane landscapes” of intracellular organelles and PMs harboring GPI-APs are typically exposed to extrinsic factors such as mechanical stress and intercellular contact. Those may cause subtle alterations in the specific configuration or assembly state of the “membrane landscapes” of PMs and organelles and thereby induce changes in their functions and (patho)physiological roles. These alterations may be transferred from mother to daughter cells or from gametes to zygotes directly along cell division and cell fusion, respectively, or indirectly in the course of vesicular and non-vesicular transfer of the constituting components of the “membrane landscapes”, including GPI-APs. Thereafter, the incorporation of newly synthesized unaltered components into the altered matrix, template, or “skeleton” of the “membrane landscapes” of PMs and organelles will lead to growth and thereby “replication” of the—eventually environmentally adapted—new versions of the “membrane landscapes”.

This (admittedly so far only theoretical) construct and thought experiment for the inheritance of acquired features by non-genetic matter should deserve experimental efforts for its proof or dismission using designs which are not imprinted by the DNS-centric view of inheritance.

## 9. Agential Realism: A Framework for Knowledge Production for (Non-Genetic) Inheritance

Drawing on the Danish physicist Niels Bohr’s study of quantum physics, the US physicist and science and technology studies (STS) scholar Karen Barad developed her theory of agential realism and challenges the representalistic idea of knowledge production [238]. This idea holds that facts, in the form of words or images, are capable of objectively reflecting pre-existing things, i.e., that representations and the objects they claim to represent are independent of one another. This implies that configurations of apparatuses or agencies of observation—i.e., the tools used in the observation of objects such as microscopes, images, gels, SAW biosensors, and computers in cellular and molecular biology—should not be framed as passive or innocent tools to peer at the object of observation and offering constraints on what the observer such as the cellular and molecular biologist can see. Rather, they should be conceived as productive and as part of the phenomenon which emerges through the process of knowledge production. That is, the measured object *and* agencies of observation form a non-dualistic physical whole. In Barad’s terminology, apparatuses are not mere observing instruments but rather material-discursive boundary drawing practices, through which differential boundaries are constituted between objects (non-humans) and subjects (humans) (for greater details of the concept of the agential realism, see Supplementary Materials, Ad 9).

We think that the meaning and potential of agential realism for the consideration or better non-consideration of the phenomenon of the intercellular transfer of GPI-APs and the apparently more or less tightly linked non-genetic inheritance of acquired biological features by both the scientific community and the public is two-fold, i.e., relevant for (i) STS as well as (ii) cellular and molecular biology and genetics.

Ad (i): STS are aimed at the practices through which the genetic trait of an inherited biological feature is produced, and the interpretative procedures which make the produced traits meaningful to the actors (e.g., parents, affected offspring) involved. What emerges from these practices is a realization that what scientists as well as the public term ‘inheritance’ is not a well-defined observation-independent object. Rather, ‘inheritance’ is a phenomenon which cannot be known—virtually does not exist—independently of apparatuses of observation applied to bring about such knowledge. Whether geneticists speak of family pedigrees, DNA markers, risk figures, or the sensations of putatively affected people, these all constitute apparatuses of observation whose measurements require interpretation before they may be acted upon [239]. If the meaning that people make of specific measurements, be they gel images, DNS marks, risk figures, SAW phase shifts or just corporeal sensations, is an element of the total phenomenon, it becomes meaningless to speak of an autonomous decision on the basis of objective facts. Rather, the whole arrangement which in total constitutes a genetic analysis may be considered as part of the emerging phenomenon, and thus as part of the decision which will be made. In future STS, the implications of the Baradian viewpoint should be explored when applied to apparatuses of the observation of biological inheritance. Those encompass experimental materials as well as empirical data and—with special emphasis—often underestimated or even neglected scientific papers, i.e., the material inscriptions or traces of the inheritance phenomenon [240] (pp. 9–29). For this, the transfer of genetic and non-genetic materials and its *intra-action* in concert with the inheritance of biological features, starting with Lamarck and Darwin’s ‘gemmules’ via the bacterial DNS transformation and epigenetics to the identification and characterization of “membrane landscapes”, including GPI-APs, and their transfer via vesicular and non-vesicular vehicles and mechanisms has to be addressed.

Ad (iia): A critical question of considerable importance will be to analyze the—presumably multiple and complex—reasons and factors leading to exclusion of the—theoretical possible—phenomenon of the biological inheritance of acquired features from being studied by researchers of life sciences as well as from being considered by politicians and the interested public. Only very recently has this open issue received some principal attention [11,241,242].

Ad (iib): Typical molecular and cell-biological apparatuses of observation will be applied to study the possibility and nature of *intra-actions* between:

- the transfer of genetic and the transfer of non-genetic materials,
- mother and daughter cells as well as soma and germline cells which do not rely on genes,
- the inheritance of features of “historical” origin, i.e., those caused by recombination/mutation prior to the start of the life of the organism and the inheritance of features acquired during the individual life span of the organism, i.e., features resulting from—intentional or non-intentional—adaptation,
- the socio-political inheritance of private property and the biological inheritance at the various stages of legislation and scientific discovery during the ages.

The fruitfulness of the consideration of technological, sociological, political, and economic factors as components of the apparatus of observation has already been demonstrated by Lily E. Kay for the discovery of the genetic code [237]. Kay did not specifically use the approach of agential realism; nevertheless, by searching in archives, published sources, and interviews, Kay managed to situate research on the genetic code (1953–1970) within the history of life sciences, the rise of communication technosciences (cybernetics, information theory, computers), the intersection of molecular biology with cryptanalysis and linguistics, and the social, political, and economic history of postwar Europe and the United States.

## 10. Conclusions

The findings summarized in this review prompt the challenging view that the transfer of biological matter in general, and of “membrane landscapes” and GPI-APs in particular, by EVs, LLPs, lipid-free aggregates, and lipid-containing micelle-like complexes represents a mode of non-genetic inheritance of phenotypes and (acquired) features. Those include the control of metabolism such as the upregulation of lipid and glycogen synthesis between mammalian somatic cells at the “sub-individual” (i.e., between cells) and “individual” (i.e., between multicellular organisms), “vertical” and “horizontal”, and “downward” and “upward” level. On the basis of the findings with iPSCs acting as efficient acceptor cells for micelle-like GPI-AP complexes, it is tempting to speculate that the transfer of matter also operates between gametes or between somatic cells and gametes. The sociological and political consequences which emerge from this concept of inheritance of matter are important. Karen Barad, one of the most important representatives of “STS”, “New Materialism”, and “Agential Realism”, stressed that during the past decades of the so-called linguistic turn the importance of language, information, and social construction has been overemphasized, while the agency of substance and matter has been underestimated or even neglected: “Language matters, Discourse matters, Culture matters. There is an important sense in which the only thing that does not seem to matter anymore is matter” [243] (p. 134). An agential-realistic study of biological inheritance at individual as well as sub-individual level which encompasses the implications for the philosophy of biology in general, and for the inscription of genetic and non-genetic traits in the scientific literature in particular, remains a desideratum for future transdisciplinary research.

## Data Availability

The datasets generated and analyzed during the current study are available from the corresponding author (G.A.M.; guenter.mueller@helmholtz-munich.de) upon reasonable request and will be provided as the original SAW data files together with the appropriate SAW Inc. software for data visualization and processing (correction and normalization), if required, under consideration of the relevant conditions for licensing of FitMaster^®^, SensMaster^®^, and SequenceMaster^®^.

## Supplementary Materials

The following supporting information can be downloaded at: https://www.mdpi.com/article/10.3390/biom13060994/s1, Figure S1. Experimental design and model for the “direct” mode of (“vertical”) transfer of GPI-APs over a short distance from human adipocytes to blood cells and stimulation of lipid droplet and glycogen granule biogenesis with the aid of transwell co-culture; complete legends and materials for Figure 2 and Figure 3; Figure S2. Upregulation of lipid synthesis in iPSCs in the course of the transfer of GPI-APs; Ad 8. The history of the concept of non-genetic inheritance of acquired features; Ad 9. Agential realism: a framework for knowledge production for (non-genetic) inheritance; Additional references. Refs. [244,245,246,247,248,249,250,251,252,253,254,255,256,257,258,259,260] can be found in Supplementary Materials.
