# Supplementary material for: (Patho)Physiology of Glycosylphosphatidylinositol-Anchored Proteins II: Intercellular Transfer of Matter (Inheritance?) That Matters
Source: Biomolecules. 2023 Jun 15;13(6):994. doi: 10.3390/biom13060994 (PMC10295932; doi:10.3390/biom13060994)
Supplement: Supplementary file 1 [file biomolecules-13-00994-s001.zip › biomolecules-2368212-supplementary.pdf]

Supplementary Materials

(Patho)Physiology of Glycosylphosphatidylinositol-Anchored Proteins II: Intercellular Transfer (Inheritance?) of Matter that Matters

Günter A. Müller and Timo D. Müller

"Direct" Mode of ("Vertical") Intercellular Transfer of GPI-APs over Short Distance

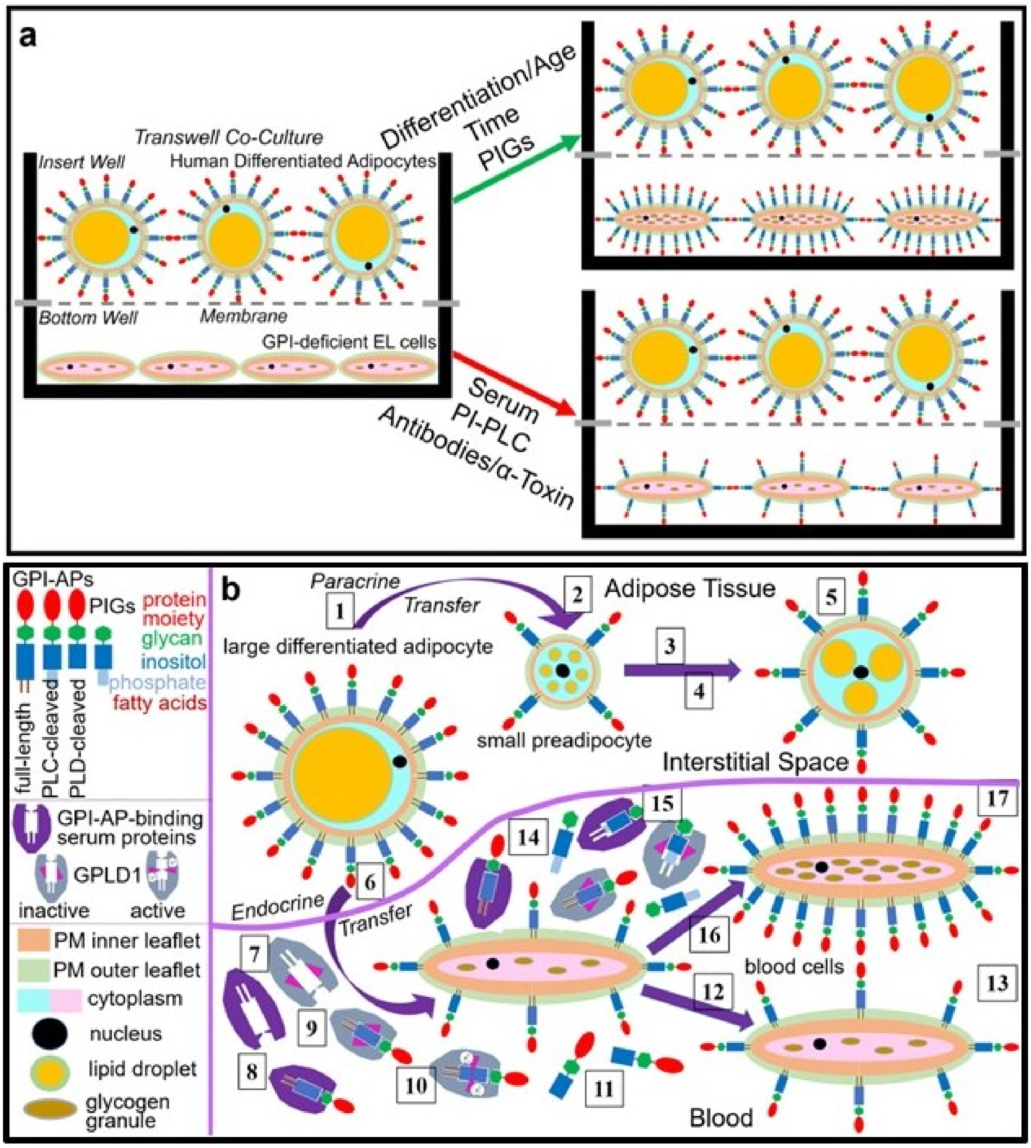

Supplementary Figure S1. Experimental design and model for the "direct" mode of ("vertical") transfer of GPI-APs over short distance from human adipocytes to blood cells and stimulation of lipid droplet and glycogen granule biogenesis with the aid of transwell co-culture.

(**a, left half**) hADSCs are seeded in top insert wells, which are equipped with a semi-permeable membrane at the bottom. During growth to confluency and differentiation into human adipocytes of varying degree of lipid-loading, GPI-deficient ELCs are seeded in a bottom well and then grown to confluency. For start of transfer, the insert wells with the human adipocytes are assembled on top of the bottom wells with the ELCs and then incubated in the presence of certain agents. GPI-AP expression at PMs (by chip-based SAW biosensing) and glycogen synthesis (by addition of U-[^{14}C]glucose) of the ELCs in the bottom plates were measured in parallel for monitoring of GPI-AP transfer and its anabolic effect.

(**a, right upper half**) Co-culture of human adipocytes in the insert wells and GPI-deficient ELCs in the bottom wells in the presence of PIGs and serum in the culture medium induces time-dependent expression of GPI-APs at the PMs of the ELCs and in parallel biogenesis of glycogen granules.

(**a, right lower half**) Transfer and anabolic effect were blocked by anti-GPI-AP antibodies or GPI-binding α-toxin, coupled to Sepharose each, bacterial PI-PLC or serum proteins in the culture medium (for explanation of the symbols, see [**b**]).

(**b**) Model for the "direct" transfer of GPI-APs between donor and acceptor cells in short distance and its consequence on the biogenesis of lipid droplets in adipose cells (upper compartment, [1-6]) and glycogen granules in blood cells (lower compartment, [7-17]) upon challenge to endogenous or exogenous cues.

Human Serum Contains GPI-Binding Proteins with Bound GPI-APs

Complete Legend and Materials for Figure 2. Human serum contains full-length GPI-APs which are bound to and displaced from serum proteins, among the GPLD1, by phosphoinositolglycans (PIGs) (for structure and design of PIGs, see [146]). (**a**) After covalent immobilization of protein A onto EDC/NHS-activated chips (0-300 sec) and subsequent blockade of unreacted carboxyl groups by ethanolamine ($EtNH_2$) (300-400 sec), anti-GPLD1 antibody (monoclonal, against human GPLD1, 1:1200, mouse IgG2a, immunogen affinity-purified and raised against a recombinant fragment corresponding to human GPLD1 aa1-300, delivered by Santa Cruz Biotechnology [Dallas, Texas, USA, D-10, sc-365096]) (blue, yellow, brown, turquoise, red curves) or IgG (green curve) were injected into the chip channels. Following washing of the chips (600-700 sec), 200 µL of human serum prepared from male AB plasma (USA origin, sterile-filtered, 40-90 mg protein/mL, 0.3 mg hemoglobin/mL and delivered by Merck/Sigma-Aldrich (Darmstadt, Germany, MDL, MFCD00165829, Cat. No. H4522) and then diluted 10-fold with PBS, containing 2 mM Pha (green curve) or lacking it (turquoise curve), which had been pretreated with bacterial PI-PLC (0.2 mU/mL, brown curve) or remained untreated (blue, yellow, turquoise, green curves) or 200 µL of PBS (red curve) were injected (700-900 sec) at a flow rate of 60 µL/min in the absence (turquoise curve) or presence of 1 mM Pha (all other curves) or 30 µM PIG41 (yellow curve). After washing of the channels with PBS (900-1000 sec) at a flow rate of 200 µL/min, α-toxin (30 µg/mL, 1000-1200 sec), and subsequently anti-CD55 (1200-1500 sec) (mouse monoclonal, protein G-purified from tissue culture supernatant, IgG2a, prepared against a recombinant fragment corresponding to the extracellular domain of human CD55; Cat. No. ab253284; 1:1000), anti-CD59 (1500-1800 sec) (rabbit monoclonal, protein A-purified, IgG isotype, prepared against a synthetic peptide corresponding to human CD59; Cat. No. ab248625; 1:2500), anti-TNAP (1800-2100 sec) (rabbit polyclonal, affinity-purified, IgG isotype, prepared against an unconjugated synthetic peptide corresponding to TNAP; Cat. No. ab954462; 1:750), anti-AChE (2100-2400 sec) (goat polyclonal, multi-step purified, IgG isotype, biotinylated, prepared against purified AChE from bovine erythrocytes; ab34533; 1:1500) and anti-CD73 (2400-2700 sec) (mouse monoclonal, prepared against CD73 purified from human placenta with no cross-reactivity against mouse/rat CD73; 1:1000; Cat. No. sc-32299) antibodies (all provided by Santa Cruz Biotechnology Inc. (Dallas, Texas, USA) were injected successively at a flow rate of 15 µL/min. After injection of 30 µM PIG41 (2700-2850 sec) together with 4 mM Ca^{2+} and then 0.2% TX-100 (2850-3000 sec) at a flow rate of 45 µL/min, anti-GPLD1 antibody (polyclonal, rabbit IgG, immunogen affinity-purified and raised against a recombinant fragment corresponding to human GPLD1 aa24-160; 1:750; purchased from Abcam Inc. [Berlin, Germany, Cat. No. ab210753]) was finally injected (3000-3300 sec) at a flow rate of 15 µL/min. The experiment was repeated once (distinct chips) with similar results (representative shown). Phase shift is given upon correction for unspecific interaction of serum components ("mock" channel lacking protein A) and altered viscosity (*vs.* buffer) of the sample fluid and normalization for the varying efficiency of distinct chips for capture of protein A. (**b**) The experiment was performed as described (see **a**; chips with immobilized anti-GPLD1) with serum prepared from untreated human male probands and diluted with Pha-containing PBS, and subsequent injection of PIG41, 37, 45, 7 and 1 (30 µM) or PBS together with Ca^{2+} (4 mM) (blue curve) and repeated once (distinct chips with four channels *per* incubation) with similar results (representative shown). The measured phase shift was corrected (see **a**) and is only shown from the

start of the last antibody (2400 sec) to the end of TX-100 injection (3000 sec). PIG-induced phase shift decreases (2600-2800 sec) are indicated (horizontal hatched lines and brackets). (**c**) After covalent immobilization (0-300 sec) of human GPLD1 (recombinant full-length protein [amino acids 1-176] with GST-tag at amino-terminus expressed in wheat germ lysate and purchased from BioMart Inc., NY, USA, Cat.No. GPLD1-5160H) onto EDC/NHS-activated in contrast to buffer-treated chips (green curve), unreacted carboxyl groups were blocked by $EtNH_2$ (300-400 sec). Following washing of the chips (400-600 sec), 200 µL of human serum (blue, yellow, brown, turquoise, green curves) prepared and delivered as described above and then diluted 10-fold with PBS, containing 2 mM Pha (blue curve) or lacking it (turquoise curve) which had been pretreated with bacterial PI-PLC (0.2 mU/mL, brown curve) or remained untreated (blue, yellow, turquoise, green curves) or 200 µL of PBS (red curve) were injected (600-900 sec) in the absence (turquoise curve) or presence of 1 mM Pha (all other curves) or 30 µM PIG41 (yellow curve). After washing of the channels with PBS (900-1000 sec) at a flow rate of 200 µL/min, α-toxin (30 µg/mL, 1000-1200 sec), and subsequently anti-CD55, anti-CD59, anti-TNAP, anti-AChE and anti-CD73 antibodies (dilutions and sources as given above) were injected successively at a flow rate of 15 µL/min. After injection of 30 µM PIG41 (2700-2850 sec) together with 4 mM Ca^{2+} and then 0.2% TX-100 (2850-3000 sec) at a flow rate of 45 µL/min, anti-GPLD1 antibody (dilution and source as given above) was finally injected (3000-3300 sec) at a flow rate of 15 µL/min. The experiment was repeated once (distinct chips) with similar results (representative shown). Phase shift is given upon correction for unspecific interaction of serum components ("mock" channel lacking protein A) and altered viscosity (vs. buffer) of the sample fluid and normalization for the varying efficiency of distinct chips for capture of GPLD1. (**d**) After covalent immobilization of full-length AChE, which had been purified from human erythrocytes according to a protocol adapted from bovine erythrocytes [244] or soluble AChE (grey curve), which had been prepared from full-length human erythrocyte AChE by digestion with bacterial PI-PLC, subsequent TX-114 partitioning and recovery from the detergent phase as described previously [245], onto EDC/NHS-activated chips (0-300 sec) and blockade of unreacted carboxyl groups by $EtNH_2$ (300-400 sec), 200 µL of human serum prepared and delivered as described above and then diluted 10-fold with PBS, containing 2 mM Pha (blue curve) or lacking it (turquoise curve) which had been pretreated with bacterial PI-PLC (0.2 mU/mL, brown curve) or remained untreated (blue, yellow, turquoise, green curves) or 200 µL of PBS (red curve) were injected (400-600 sec) in the absence (turquoise curve) or presence of 1 mM Pha (all other curves) or 30 µM PIG41 (yellow curve). After washing of the channels with PBS (600-700 sec), monoclonal anti-GPLD1 antibody (dilution and source as described above) or IgG (green curve) (700-900 sec), human annexin-V (100 µg/mL, recombinant full-length GST-tagged protein expressed in *E. coli* and delivered by Sigma-Aldrich/Merck, Darmstadt, Germany, Cat.No. SRP8026) (900-1000 sec), α-toxin (30 µg/mL, 1000-1200 sec), and subsequently anti-CD55 (1200-1500 sec), anti-CD59 (1500-1800 sec), anti-TNAP (1800-2100 sec), and anti-CD73 (2100-2400 sec) antibodies (dilutions and sources as described above) were injected successively at a flow rate of 15 µL/min. After injection of 0.2% TX-100 (2400-2600 sec) and then 30 µM PIG41 together with 4 mM Ca^{2+} (2600-2800 sec) at a flow rate of 40 µL/min, anti-AChE antibody (2800-2900 sec, goat polyclonal, multi-step purified, IgG isotype, biotinylated, prepared against purified AChE from bovine erythrocytes; 1:1500; purchased from Abcam Inc. [Berlin, Germany, ab34533]) at a flow rate of 30 µL/min, then 250 µL of PBS (2900-3000 sec) at a flow rate of 150 µL/min and finally GPLD1 (30 µg/mL, source as described above, 3000-3300 sec) together with 1 mM Pha at a flow rate of 15 µL/min were injected. The experiment was repeated once (distinct chips) with similar results (representative shown). Phase shift is given upon correction for unspecific interaction of serum components ("mock" channel lacking protein A) and altered viscosity (*vs*. buffer) of the sample fluid and normalization for the varying efficiency of distinct chips for capture of AChE.

GPI-APs Are Transferred from Human Adipocytes to iPSCs during Transwell Co-Culture

Complete Legend and Materials for Figure 3 (according to ref. [143] with the following modifications**)**. Full length GPI-APs are transferred from human differentiated cells, human micelle-like complexes and human serum proteins to human induced pluripotent stem cells (iPCSs) under the control of PIGs, insulin and antidiabetic sulfonylureas (SUs). (**a**) Transfer of full-length GPI-APs from human adipocytes to human iPSCs. Transwell co-cultures were run with untreated human adipocytes of lipid loading stage II as donor cells (A) or medium alone (M) and mannosamine (ManN)-treated human iPSCs as acceptor cells in the insert and bottom wells. Human iPSCs (depositor Well Trust

Sanger Institute) are derived from fibroblasts of dermis of the arm of normal male adult probands (primary cell type) using non-integrating Sendai virus (KLF4, MYC, POU5F1, SOX2) and displayed the following marker expression TRA1-60(+),NANOG(+),SOX2(+),POU5F1,SSEA-1(-),SSEA-4(+) with a pluripotency score of 22.015. The human iPS cell line WTSli146-A (from the EBiSC stem cell bank / Fraunhofer-Gesellschaft, passage number 17, karyotype 46, XY) was purchased from Sigma-Aldrich/Merck (Darmstadt, Germany, Cat.No. 66540257) after having signed the EBiSC Access Use Agreement. Adherent iPSCs were cultured using vitronectin-coated tissue plates, enzyme-free cell dissociation / EDTA, culture medium TeSRE8 (WiCell Research Institute Inc., USA) and 5% CO_2. After incubation (37 °C) for various times (h, hours; d, days; and w, weeks), PMs were prepared from the cells of the bottom wells, coupled to chips by ionic / covalent capture and then analyzed for expression of the membrane proteins indicated by surface acoustic waves (SAW) sensing. Phase shifts induced by binding of antibodies against GPI-APs (source and dilution, see above) or transmembrane proteins (anti-Glut1; rabbit polyclonal, generated against a synthetic peptide corresponding to rat Glut1 carboxy-terminus aa 481-492, immunogen affinity-purified, IgG isotype, 1:200 and anti-Glut4; rabbit polyclonal, generated against a synthetic peptide corresponding to human Glut4 carboxy-terminus aa 1-450 and conjugated to KLH, immunogen affinity-purified, IgG isotype, 1:500 were purchased from Abcam, Berlin, Germany, Cat.No. ab14683 and ab33780, respectively) and by PI-PLC- and TX-100-treatment (from 700 to 3100 sec) are shown, only, omitting the preceding capturing procedures (0–700 sec). Correction and normalization of the phase shift were performed as described in the legend to Figure 2. Phase shift Δ between injection of the first (at 800 sec) and last (2700 sec) antibodies against GPI-APs are indicated by horizontal hatched lines and brackets for each incubation condition ([A] donor adipocytes, [M] medium alone in the inset wells) and period. PMs from representative cell clones/preparations analyzed by different chips are shown, repeated four times with similar results. (**b**) Transfer of full-length GPI-APs from human micelle-like GPI-AP complexes to iPSCs. ManN-treated human iPSCs were incubated with buffer (Control) or the indicated amounts of human adipocyte micelle-like GPI-AP complexes (prepared as described previously [142]) for the indicated periods. PMs were prepared from the iPSCs of the bottom wells, coupled to chips by ionic (+ Ca^{2+}) / covalent (EDC/NHS) capture and then, following blockade of the remaining reactive groups by $EtNH_2$, removal of unbound PMs by EGTA and washing of the chip channels with NaCl and buffer, analyzed for expression of the membrane proteins indicated by SAW biosensing as described for (**a**). The measured phase shift is given upon correction and normalization as described for Figure 2. PMs from representative cell clones/preparations analyzed by different chips are shown, repeated three to four times with similar results. (**c**) Transfer to human iPSCs of GPI-APs released from human serum proteins by PIG41. ManN-treated human iPSCs were incubated (1 week, 37 °C, 5 mM glucose) in the bottom wells of transwell co-cultures with 200 μL buffer or serum, which had been prepared from normal human probands, then diluted 10-fold with PBS lacking or containing 2 mM ortho-phenanthroline (Pha) and thereafter left untreated or incubated with proteinase K (PK), bacterial PI-PLC (PLC), α-toxin coupled to Sepharose beads (α-toxin) or 30 μM PIG41 as indicated. Thereafter, the iPSCs were washed thoroughly by rinsing the bottom wells three times with 2 mL each of 20 mM Tris/HCl, 1.5 M NaCl and then two times with 2 mL each of PBS. Subsequently, PMs were prepared from the iPSCs, coupled to chips by ionic/covalent capture (0-200 sec), as described previously [133], and then analyzed for expression of GPI-APs by SAW sensing. As a control, 200 μL serum from normal human probands instead of PMs were injected into the chips (0-200 sec) at a flow rate of 60 μL/min (a, brown curve). After washing of the channels with buffer (200-300 sec) at a flow rate of 200 μL/min, α-toxin (30 μg/mL, 300-500 sec), anti-CD55 (500-800 sec), anti-CD59 (800-1100 sec), anti-TNAP (1100-1400 sec), anti-AChE (1400-1700 sec) and anti-CD73 (1700-1950 sec) antibodies (at the dilutions given above) were injected successively at a flow rate of 15 μL/min, followed by 30 μM PIG41 (1950-2150 sec) and then 0.2% TX-100 (2150-2300 sec) at a flow rate of 45 μL/min. Measured phase shifts were corrected and normalized. Phase shift Δ between start of the α-toxin (300 sec) and termination of the anti-CD73 antibody (2000 sec) injection indicated by horizontal hatched lines are given by brackets. (**d**) Restoration of insulin- and SU-inhibited GPI-AP transfer by serum. Transwell co-cultures were run with human adipocytes (of lipid-loading stage II) as donor cells or only medium (control no transfer) and human ManN-treated iPSCs as acceptor cells in the insert and bottom wells, respectively, as described previously. The co-cultures were incubated (37 °C, 1 week, 5 mM glucose) in the absence (control transfer) or presence of human insulin (1 or 30 nM; ins) or glimepiride (3 or 30 μM; gli) without or with 200 μL of untreated serum, which had been prepared from normal human probands and then

diluted 10-fold with PBS containing 2 mM Pha, in the presence of Pha (1 mM). Thereafter PMs were prepared from the iPSCs of the bottom wells, coupled to chips by ionic/covalent capture and then analyzed for expression of membrane proteins by SAW sensing as described for (**a**). Phase shifts induced by binding of antibodies against membrane proteins (800-2700 sec) and by subsequent PI-PLC- and TX-100-treatments (2700-3200 sec) are shown, only, omitting the preceding capturing of the PM (0-700 sec). Correction and normalization of the phase shift Δ were performed as described for (**a**). The experiment was repeated three to six times using serum from different human normal probands, with only a representative shown. Phase shift Δ between start of injection of anti-CD55 antibody (at 800 sec) and termination of injection of anti-AChE antibody (at 2650 sec) are indicated by horizontal hatched lines and brackets for each incubation condition.

Transfer of GPI-APs to Human iPSCs Induces Lipid Synthesis

The iPSCs which had been analyzed as acceptor cells for transfer of GPI-APs from human donor adipocytes (see Figure 2a,b) as well as its control by various agents, serum, insulin and glimepiride (see Figure 2c,d) are studied for lipid synthesis to elucidate a putative role of transfer in determining the metabolic phenotype (Supplementary Figure S2).

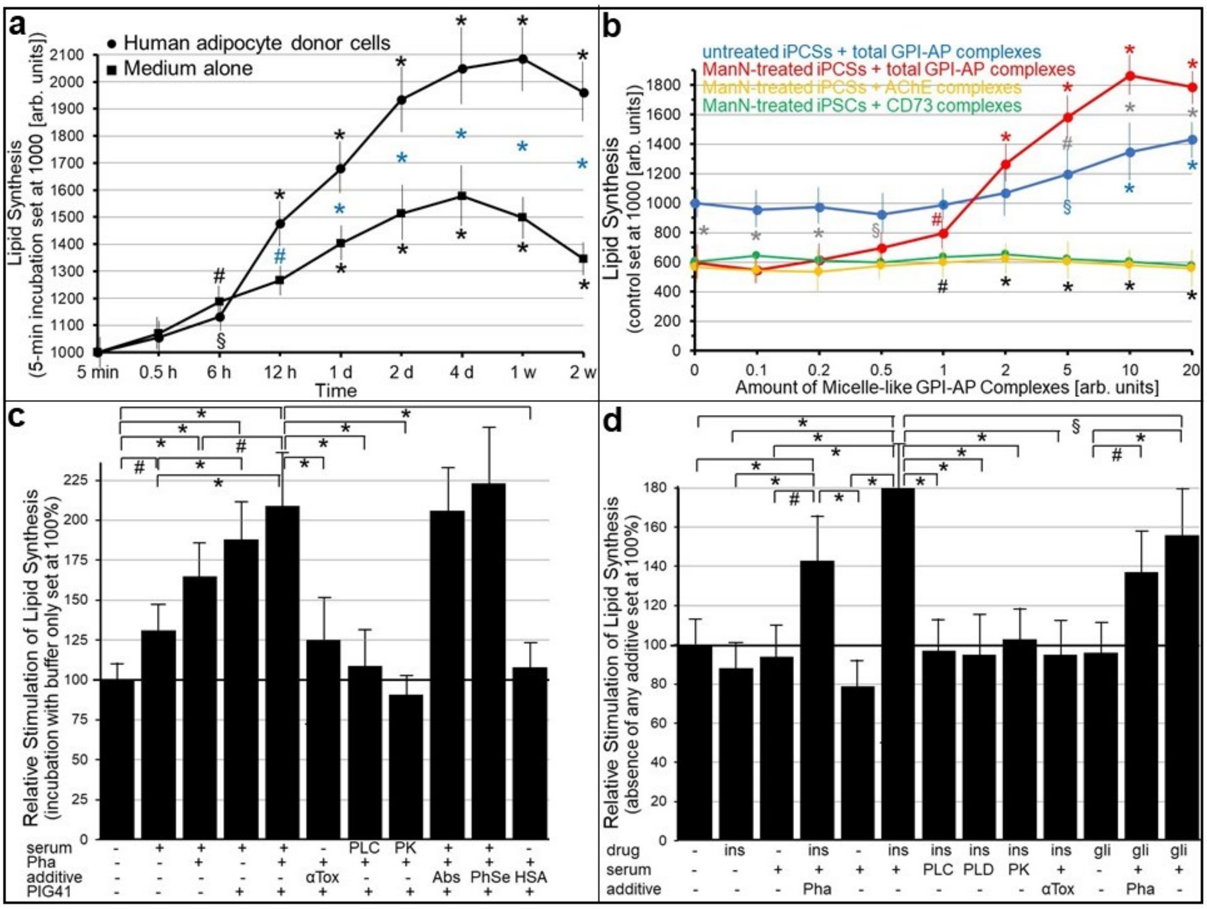

Supplementary Figure S2. Upregulation of lipid synthesis in iPSCs in course of transfer of GPI-APs. (**a**) ([143] see Fig. 10d) Lipid synthesis in iPSCs upon co-culturing with human adipocytes. Transwell co-cultures were run with untreated human adipocytes of lipid loading stage II as donor cells (circles) or medium alone (squares) and mannosamine (ManN)-treated human iPSCs as acceptor cells in the insert and bottom wells, respectively. After the periods of incubation indicated, iPSCs were assayed for lipid synthesis by measurement of the incorporation of fluorescent fatty acids into total acylglycerols during 30 min of incubation (37 °C) with their subsequent separation by thin layer chromatography and quantitative evaluation by fluorescence imaging as described previously [133,246]. Mean values ± SD (five to seven independent incubations and assays, each) of the synthesized acylglycerols are given as arb. units set at 1000 for incubation (5 min). Significant differences for each vs. 5-min incubation as well as for donor cells vs. medium at each incubation time are indicated (black and blue symbols, respectively). (**b**) ([143] see Figure 7c). Lipid synthesis in iPSCs

upon incubation with micelle-like GPI-AP complexes. Untreated (blue line) or ManN-treated (green, orange, red line) human iPSCs were incubated (30 min, 37 °C) without (control) or with increasing amounts of micelle-like GPI-AP complexes, which had been reconstituted from total human adipocyte GPI-APs (red line) or purified CD73 (green line) or AChE (orange line) as described previously [133]. Thereafter the iPSCs were assayed for lipid synthesis as described above for (a). The mean values ± SD (three to seven independent incubations and assays, each) of the synthesized lipids are given as arb. units set at 1000 for incubation of untreated iPSCs without complexes. Significant increases for each amount of complexes vs. their absence are indicated by colored symbols. Significant differences between total GPI-AP and AChE complexes as well as between untreated and ManN-treated IPSCs incubated with total GPI-AP complexes are indicated by black and grey symbols, respectively. (**c**) ([133] see Figure 11a) Lipid synthesis in iPSCs upon incubation with serum proteins and PIGs. ManN-treated human iPSCs were incubated (30 min, 37 °C) in the bottom wells of transwell co-cultures with 200 μL of PBS (buffer) or serum which had been obtained from normal human probands, then diluted 10-fold with PBS lacking or containing 2 mM Pha, or human serum albumin (HSA; purity ≥ 98%, Sigma-Aldrich/Merck, Darmstadt, Cat.No. SRP6182) (4 mg/mL PBS) in the presence of 4 mM Ca^{2+} or 1 mM Pha (1 mM) as indicated and subsequently left untreated (serum) or incubated with proteinase K (PK), bacterial PI-PLC (PLC) or supplemented with α-toxin (αTox) or antibodies against TNAP, CD73 and AChE (Abs), each coupled to Sepharose beads, or phenyl Sepharose beads (PhSe) prior to addition to the transwell co-cultures, in the absence or presence of PIG41 (30 μM). Thereafter, the iPSCs were washed intensely by rinsing the bottom wells three times with 2 mL each of 20 mM Tris/HCl, 1.5 M NaCl and then two times with 2 mL each of PBS and subsequently assayed for lipid synthesis (30 min, 37 °C) as described for (a). The incubations were repeated four to six times with determination of lipid synthesis in quadruplicate. Relative stimulation of lipid synthesis by serum and PIG41 in the presence of Pha or other additives is given with incubation with buffer only set at 100%. (**d**) ([133] see Figure 8a) Lipid synthesis in iPSCs upon incubation with serum, insulin and SUs. Transwell co-cultures were run with untreated human adipocytes of lipid loading stage II as donor cells and ManN-treated human iPSCs as acceptor cells in the insert and bottom wells, respectively, as described for (a), in the absence or presence of human insulin (ins, 30 nM) or glimepiride (glim, 30 μM) without or with 200 μL serum, which had been prepared from normal human probands and then diluted 10-fold with PBS containing 2 mM Pha, in the presence of 4 mM Ca^{2+} or 1 mM Pha as indicated. The serum had been left untreated or digested with bacterial PI-PLC (PLC), human GPLD1 (PLD) or proteinase K (PK) or supplemented with α-toxin coupled to Sepharose beads (αTox) prior to addition to the transwell co-cultures. Thereafter, the iPSCs were assayed for lipid synthesis (30 min, 37 °C) as described for (a). The transwell co-cultures were repeated four to six times (distinct transwell co-cultures) with determination of lipid synthesis in triplicate. Relative lipid synthesis is given with absence of any additive set at 100% (horizontal black line). Significant differences are indicated (means ± S.D.; * $p<0.01$, # $p< 0.02$, § $p < 0.05$).

Transwell co-culture of differentiated human adipocytes with undifferentiated ManN-treated human iPSCs led to stimulation of lipid synthesis in the latter (Supplementary Figure S2a, circles) compared to incubation with medium alone (Supplementary Figure S2a, squares), with significant differences measurable from 12 h to 2 weeks of co-culturing time. The failure of a transwell co-culture with 0.4 μm pore size to support transfer and lipid synthesis stimulation (Müller and Müller, data not shown) made the involvement of soluble entities other than full-length GPI-APs released from the donor cells, such as cytokines [67-70], in altering the metabolic phenotype of iPSCs unlikely. This correlation between GPI-AP transfer and upregulated lipid synthesis suggested a role of the transferred GPI-APs in the control of the metabolic phenotype in human iPSCs.

To corroborate this possibility, human adipocytes and iPSCs were incubated with micelle-like complexes reconstituted with total human adipocyte GPI-APs or purified human CD73 or AChE in the absence of serum and then assayed for synthesis of lipids (in the presence of BSA). In ManN-treated iPSCs lipid synthesis was upregulated by total GPI-AP complexes in concentration-dependent fashion to up to 3.2-fold above basal (no complexes) (Supplementary Figure S2b, red line). In contrast, in untreated iPSCs lipid synthesis was only increased by 1.4-fold at the highest amount of complexes (Supplementary Figure S2b, blue line). Importantly, complexes with human CD73 and AChE were completely inactive in both ManN-treated (Supplementary Figure S2b, green

and orange line) and untreated (data not shown, Müller and Müller, manuscript in preparation) iPSCs. These data strongly suggested that transfer of GPI-APs from micelle-like complexes reconstituted with total human adipocyte GPI-APs, rather than solely with human CD73 or AChE, to human iPSCs with downregulated PM expression of GPI-APs stimulates lipid synthesis.

The above data raised the possibility that lipid synthesis is stimulated in iPSCs upon exposure to serum proteins with bound full-length GPI-APs under conditions which trigger their dissociation. In fact, ManN-treated human iPSCs incubated with serum from healthy male probands in the absence of Pha caused significant stimulation of lipid synthesis which was further increased by the presence of Pha during preparation of the serum, presence of PIG41 and, most potently, by the two conditions in combination (Supplementary Figure S2c). The elevated lipid synthesis was reduced by the presence of α-toxin Sepharose beads during incubation of the serum with the cells, or pretreatment of the serum with bacterial PI-PLC or proteinase K. At contrast, a combination of anti-CD55, CD59, TNAP, CD73 and AChE antibodies coupled to Sepharose beads or phenyl Sepharose beads present during incubation of the serum with the iPSCs did not affect stimulation of lipid synthesis by PIG41 and serum, which had been prepared in the presence of Pha (Supplementary Figure S2c). Finally, human serum albumin (HSA) failed to substitute for serum in upregulating lipid synthesis in iPSCs. This was explained best with stimulation of lipid synthesis in iPSCs with diminished GPI-AP expression in course of transfer of full-length GPI-APs from human serum proteins to their PMs. However, transferred CD55, CD59, TNAP, CD73 and AChE apparently have no effect on lipid synthesis. Importantly, lipid synthesis stimulation was most pronounced upon simultaneous inhibition of serum GPLD1 and stabilization of the interaction of GPI-APs with the serum binding-proteins by missing Ca^{2+} during serum preparation in combination with displacement of the GPI-APs from the binding-proteins by PIG41 during subsequent serum injection in the presence of Ca^{2+}.

Finally, it was investigated whether abrogation of the insulin or SU inhibition of the "direct" transfer of GPI-APs from human adipocytes to iPSCs by human serum leading to "indirect" transfer (see Supplementary Figure S2d) is reflected in upregulation of lipid synthesis in the iPSCs. To test for this, human adipocytes as donor cells and iPSCs with impaired GPI-AP expression as acceptor cells were incubated in transwell co-culture in the presence of insulin (ins) or glimepiride (gli) without or with human serum, which had been pretreated under various conditions to get rid of (full-length) GPI-APs (Supplementary Figure S2d). Assaying of the iPSCs for basal lipid synthesis (5 mM glucose) revealed that each serum, insulin and glimepiride alone did not exert any significant effect. At contrast, insulin or glimepiride in combination with human serum significantly stimulated lipid synthesis with the former being more potent than the latter, which both were diminished by Pha. Lipolytic (by PI-PLC and GPI-PLD) and proteolytic (by PK) pretreatment of human serum as well as α-toxin Sepharose beads (αTox) completely blocked serum-stimulated lipid synthesis in the presence of either insulin or glimepiride (Supplementary Figure S2d). Strikingly, antibodies against TNAP, CD73 and AChE coupled to Sepharose beads as well as HSA had no effect on lipid synthesis compared to the corresponding control (Müller and Müller, data not shown).

Ad 8. History of the Concept of Non-Genetic Inheritance of Acquired Features

The inheritance of biological features is a commonsense concept. Human beings have known for a long time of the existence of many reliable similarities between parents and offspring. Therefore, for an equally long time, they must have had a concept that refers to the process – whatever they are – responsible for these reliable similarities. And they must have had concepts that refer – either in a backward looking way (i.e. inherited) or in a forward-looking way (i.e. heritable) to the features that reliable reoccur within lineages thanks to these processes. This like-begets-like phenomenon belongs to the most broadly accepted biological phenomena, discussed in some of the oldest scientific treatises about biological matter. The like-begets-like phenomenon can be explained by appealing exclusively to what happens at the moment of "donation" and "conception": Some

substances are "donated" by the mother cells or gametes which upon "conception" by the daughter cells and zygotes, respectively, ultimately will lead to a new organism. Those substances carry within some matter (or principle or factor) that becomes part of the new organism and causes it to acquire some of the features of the mother cells or parents, including both features that the mother cells or parents share with the other cells of individuals of their type or species and features that are specific to the mother cells and parents, respectively. What happens is that the mother cells or parents at "conception" "donate" to their daughter cells or offspring some developmentally important matter that causes the daughter cells or offspring to develop in such a way as to resemble the mother cells or the parents, respectively. All the theories of the like-begets-like phenomenon that have this structure may be called "donation/conception" theories.

With very few exceptions, all the theories of the like-begets-like phenomenon ever formulated are of the "donation/conception" type. For example, the 17th century supporters of the theory of preformation as well as of the theory of epigenesis held a "donation/conception" view. The difference between them was the nature of the matter "donated" by mother cells or parents to daughter cells and offspring, respectively, at "conception", but there was agreement about the assumption that the like-begets-like phenomenon had to be explained by the transfer of this matter from mother cells or parents to daughter cells or offspring. According to the supporters of preformation, the matter transferred from parents to offspring (and contained in sexual substances) was constituted by miniature versions of the organisms and their features. According to the supporters of epigenesis, the matter contained some building blocks, from which the new organism "unfolds", i.e. develops, rather than miniature versions of it. But the supporters of both theories agreed about the view that the crucial elements for the explanation of the like-begets-like phenomenon were to be found in the transferred matter [247,248].

Another example comes from the comparison of the theories of the like-begets-like phenomenon formulated by Lamarck, Darwin, and Weismann. All three researchers of inheritance, development and evolution held a "donation/conception" theory. Their disagreement was about whether the like-begets-like phenomenon extended to new idiosyncratic features that organisms acquire during their life or not. Jean-Baptiste de Lamarck [249] and Charles Darwin [12] thought that it did. August Weismann thought that it did not [250]. Darwin tried to give a mechanistic explanation of how the like-begets-like phenomenon could apply to 'acquired characters' [12,13]. This is his famous theory of "pangenesis", which was rejected by Weismann [251]. But there was no disagreement about the "donation/conception" theory. In fact, both Darwin's and Weismann's arguments presuppose the "donation/conception" theory and those would not make sense in a context where this theory is not taken as granted. In order to argue that the like-begets-like phenomenon applies to 'acquired characters', Darwin argued that such characters could be passed on to the offspring in this way [13]: The 'acquired characters' produce so-called 'gemmules', which are then donated or transferred to the offspring at conception and here cause the development of features similar to those by which the 'gemmules' have been produced in the parents. Darwin was assuming that only a mechanism that explains the transfer of acquired parental features through the transfer at conception of some special matter could be scientifically acceptable. In contrast, Weismann claimed that nothing like the Darwinian 'gemmules' could exist [250]. From this he inferred that acquired parental features could not be passed on to the offspring through the transfer of some matter at conception. And from this he inferred that no mechanism for passing on these features could exist.

It is because of the attractiveness of the "donation/conception" theory that the metaphor of inheritance was formulated and adopted – originally from the social and political context and presumably very early in the history of mankind, during the neolithic revolution with the introduction of agriculture and cattle-breeding as well as monogamy. And it is because of its plausibility that the metaphor of inheritance became entrenched. But it is important to keep in mind that one can support the concept of the inheritance of biological features without having any specific

view about the nature of this process, in general, and the nature of the matter transferred, in particular.

Ad 9. Agential Realism: A Framework for Knowledge Production about (Non-Genetic) Inheritance

Drawing on the Danish physicist Niels Bohr's study of quantum physics, the US physicist and science and technology studies (STS) scholar Karen Barad developed her theory of agential realism and challenges the representalistic idea of knowledge production [238]. This idea holds that facts, in the form of words or images, are capable of objectively reflecting pre-existing things, i.e. that representations and the objects they claim to represent are independent of one another. These views have been challenged by several science studies and STS scholars [252-255]. Although these scholars differ in significant ways, attention has been drawn to how knowledge production must be analyzed as a process through which both human and non-human forms of agency are involved, and that the coming together of the human and the non-human evolves through a process of co-production [256,257]. Likewise, Barad's theory of agential realism starts out from the premise that 'we need to understand in an integral way the roles of human and nonhuman, material and discursive, and natural and cultural factors in scientific and other practices' ([243] see p.25).

Barad goes further, however, than the mere acknowledgement that material and human agency play a role in knowledge production. Her starting point is Niels Bohr's demonstration that classical correspondence theories of scientific knowledge fall short when trying to explain the classical wave-particle paradox; that light manifest particle-like properties under one set of experimental circumstances and wave-like properties under a different set of experimental circumstances ([243] see p.198) [258]. To a classical realist, who sees the relationship between scientific knowledge and the object under observation as a relationship of correspondence or mirroring, this situation is paradoxical: 'The true ontological nature of light is in question; either light is a wave, or it is a particle; it cannot be both' ([243] see p.198). Bohr resolves this problem, in Barad's interpretation, by installing 'phenomena' understood as the wholeness of the entity of observation and the apparatus of observation as the referent of knowledge. Bohr seemingly denied the duality of an independent observer and an observation-independent object [258].

Although Bohr's theory is concerned with physical matter, Barads shows how his thinking can be made productive in a social and biological science context. Taking into account Bohr's notion of a phenomenon, Barad develops the concept of *intra-action* which underscores the sense in which entities emerge through their encounters with each other in a continual process of becoming ([243] see p.33). *Intra-action* is opposed to the concept of interaction, which is very common and has already been very successful in the areas of molecular genetics and biology. Interaction is based on the pre-existence of two distinct – biological – entities given in advance that come together and engage in some kind of exchange, such as of a specific three-dimensional structure. At variance, Barad suggests that distinct entities, be it human and/or non-human, the – social – discursive and/or the biological material do not precede practice. Rather, the entities are only distinct in relation to their mutual entanglement, i.e. they do not exist as individual "pre-existing" elements ([243] see p.33).

This implies that configurations of apparatuses or agencies of observation – i.e. the tools used in the observation of objects, such as microscopes, images, gels, SAW biosensor, computers, in cellular and molecular biology – should not be framed as passive or innocent tools to peer at the object of observation of offering constraints on what the observer, such as the cellular and molecular biologist, can see. Rather, they should be conceived as productive and part of the phenomenon which emerges through the process of knowledge production. That is, the measured object *and* agencies of observation form a non-dualistic physical whole: 'Phenomena are the ontological inseparability of agentially intra-acting components' ([243] see p.33). It is this relational and multidimensional entity that comprises the phenomenon ([243] see p.205). On that basis, Barad developed what she calls a non-representationalist – but nevertheless also non-constructivist (in its social meaning) and still realistic – account of scientific practices that takes the *material* nature of

knowledge practices seriously. In Barad's terminology apparatuses are not mere observing instruments but rather material-discursive boundary drawing practices, through which differential boundaries are constituted between objects (non-humans) and subjects (humans) ([243] see p. 140). Barad's work goes further than theories of co-production in its attempt to create a more robust understanding of materiality. Thereby she managed successfully to integrate a notion of the nature/the material as fundamental for knowledge production without reinstalling it as something which is an outside or a pre-existing practice.

We find Barad's approach challenging in the analysis of the way that human and non-human *intra-actions* constitute knowledge production but also in her way of explicitly drawing out the ontological, ethical, and political implications. If we acknowledge that the basic units of reality are phenomena, understood as the ontological inseparability of emergent configurations of (human and non-human) agencies, then we are in part responsible for what there 'is' in the world: 'We are responsible for the world on which we live not because it is an arbitrary construction of our choosing, but because agential reality is sedimented out of particular practices that we have a role in shaping' ([243] see p.247). Following this, Barad suggested that ethical concerns must not simply be considered as supplemental to the practices of science [259,260]. This has been and still is the case in genetics studies on the inheritance of human diseases, where ethical concerns are centered on questions of responsibility, such as how to produce objective predictions of the disease risk and how to make possible non-directive communication with regard to consequences for the affected individuals. Rather, ethical concerns must be considered as an integral part of knowledge production and its consequences. This specific point will be addressed in a forthcoming study (Müller, manuscript in preparation).

References

12. Darwin, C. Pangenesis: Mr. Darwin's reply to Professor Delpino scientific opinion. *A Weekly Record of Scientific Progress at Home & Abroad*, **1869**, p. 426.

13. Darwin, C. Pangenesis. *Nature* **1871**, *3*, 502.

67. Rondinone, C.M. Adipocyte-derived hormones, cytokines, and mediators. *Endocrine* **2006**, *29*, 81-90.

68. Maslowska, M.; Wang, H.W.; Cianflone, K. Novel roles for acylation stimulating protein/C3ades/Arg: a review of recent *in vitro* and *in vivo* evidence. *Vitam. Horm.* **2005**, *70*, 309-332.

69. Chou, W.L.; Chuang, L.M.; Chou, C.C.; Wang, A.H.; Lawson, J.A.; FitzGerald, G.A.; Chang, Z.F. Identification of a novel prostaglandin reductase reveals the involvement of E2 catabolism in regulation of peroxisome proliferator-activator receptor gamma activation. *J. Biol. Chem.* **2007**, *282*, 18162-18172.

70. Yu, Y.H.; Chang, Y.C.; Su, T.H.; Nong, J.Y.; Li, C.C.; Chuang, L.M. Prostaglandin reductase-3 negatively modulates adipogenesis through regulation of PPARγ activity. *J. Lipid Res.* **2013**, *54*, 2391-2399.

133. Müller, G.A.; Ussar, S.; Tschöp, M.H.; Müller, T.D. Age-dependent membrane release and degradation of full-length glycosylphosphatidylinositol-anchored proteins in rats. *Mech. Ageing Dev*. **2020**, *190*, 111307.

142. Müller, G.A.; Tschöp, M.H.; Müller, T.D. Upregulated phospholipase D activity toward glycosylphosphatidylinositol-anchored proteins in micelle-like serum complexes in metabolically deranged rats and humans. *Am. J. Physiol. Endocrinol. Metabol.* **2020**, *318*, E462-E479.

143. Müller, G.A.; Müller, T.D. Transfer of proteins from cultured human adipose to blood cells and induction of anabolic phenotype are controlled by serum, insulin and sulfonylurea drugs. *Int. J. Mol. Sci.* **2023**, *24*, 4825.

146. Frick, W.; Bauer, A.; Bauer, J.; Wied, S.; Müller, G. Structure-activity relationship of synthetic phosphoinositolglycans mimicking metabolic insulin action. *Biochemistry* **1998**, *37*, 13421-13436.

238. Barad, K. Agential realism: How material-discursive practices matter. *Signs* **2003**, *28*, 803-831.

243. Barad, K. Meeting the universe halfway. *Duke University Press*, **2007**, Durham, London.

244. Berman, J.D.; Young, M. Rapid and complete purification of acetylcholinesterases of electric eel and erythrocyte by affinity chromatography. *Proc. Natl. Acad. Sci. USA* **1971**, *68*, 395-398.

245. Toutant, J.P.; Roberts, W.L.; Murray, N.R.; Rosenberry, T.L. Conversion of human erythrocyte acetylcholinesterase from an amphiphilic to a hydrophilic form by phosphatidylinositol-specific phospholipase C and serum phospholipase D. *Eur. J. Biochem*. **1989**, *180*, 503-508.

246. Müller, G.; Jordan, H.; Petry, S.; Wetekam, E.-M.; Schindler, P. Analysis of lipid metabolism in adipocytes using fluorescent fatty acids. I. Insulin stimulation of lipogenesis. *Biochim. Biophys. Acta* **1997**, *1347*, 23-39.

247. Pinto Correia, C. The Ovary of eve: Egg and sperm and preformation. *University of Chicago Press*, **1997**, Chicago.

248. Benson, K. Epigenesis. In: Pagel, M. (ed.), The Oxford encyclopedia of evolution. *Oxford University Press*, **2002**, Oxford.

249. De Lamarck, J.B. Philosophie Zoologique, **1809**.

250. Weismann, A. Essays upon heredity and kindred biological problems. *Oxford University Press*, **1989**, Oxford.

251. Weismann, A. Das Keimplasma: Eine Theorie der Vererbung. *Gustav Fischer*, **1892**, Jena.

252. Collins, H.M.; Yearly, S. Epistemological chicken, in: Pickering, A. (ed.); *Science as Practice and Culture*, University of Chicago Press, **1992**, Chicago, London, pp. 301-326.

253. Hacking, I. Representing and intervening. *Cambridge University Press*, **1983**, Cambridge**.**

254. Pickering, A. The mangle of practice: Time, agency, and science. *University of Chicago Press*, **1995**, Chicago.

255. Rouse, J. Engaging science: How to understand its practices philosophically. *Cornell University Press*, **1996**, Itacha, New York.

256. Callon, M.; Latour, B. Don't throw the baby out with the bath school! A reply to Collins and Yearly, in: Pickering, A. (ed.); *Science as Practice and Culture*, University of Chicago Press, **1992**, Chicago, London, pp. 343-368.

257. Latour, B. We have never been modern. *Harvard University Press*, **1993**, Cambridge.

258. Bohr, N. Atomic physics and human knowledge. *John Wiley and Sons Inc*., **1958**, New York.

259. Barad, K. Getting real: technoscientific practices and the materialization of reality. *Differences: J. Feminist Cult. Stud.* **1998**, *10*, 87-126.

260. Barad, K. Posthumanist performativity: how matter comes to matter. *Signs: J. Women Cult. Sci.* **2003**, *28*, 801-831.
